# ^1^H nuclear magnetic resonance-based metabolite profiling of guava leaf extract: an attempt to develop a prototype for standardization of plant extracts

**DOI:** 10.1186/s12906-021-03221-5

**Published:** 2021-03-18

**Authors:** Manasi S. Gholkar, Jia V. Li, Poonam G. Daswani, P. Tetali, Tannaz J. Birdi

**Affiliations:** 1 Department of Medicinal Plants, The Foundation for Medical Research, Dr. Kantilal J. Sheth Memorial Building, 84-A, R.G, Thadani Marg, Worli, Mumbai - 400018, MAHARASHTRA India; 2 Department of Metabolism, Digestion and Reproduction, Faculty of Medicine, Imperial College London, London, UK; 3 Presently Freelance Consultant & Formerly Scientist at Naoroji Godrej Centre for Plant Research (NGCPR), Shirwal, Maharashtra India

**Keywords:** ^1^H NMR, Metabolomics, *Psidium guajava*, India

## Abstract

**Background:**

Herbal medicines are fast gaining popularity. However, their acceptability by modern practitioners is low which is often due to lack of standardization. Several approaches towards standardization of herbals have been employed. The current study attempted to recognize key peaks from ^1^H NMR spectra which together would comprise of a spectral fingerprint relating to efficacy of *Psidium guajava* (guava) leaf extract as an antidiarrhoeal when a number of unidentified active principles are involved.

**Methods:**

Ninety samples of guava leaves were collected from three locations over three seasons. Hydroalcoholic (water and ethanol, 50:50) extracts of these samples were prepared and their ^1^H NMR spectra were acquired. Spectra were also obtained for quercetin, ferulic acid and gallic acid as standards. Eight bioassays reflecting different stages of diarrhoeal pathogenesis were undertaken and based on pre-decided cut-offs, the extracts were classified as ‘good’ or ‘poor’ extracts. The bioactivity data was then correlated with the ^1^H NMR profiles using Regression or Orthogonal Partial Least Square-Discriminant Analysis (OPLS-DA).

**Results:**

OPLS-DA showed seasonal and regional segregation of extracts. Significant models were established for seven bioassays, namely those for anti-bacterial activity against *Shigella flexneri* and *Vibrio cholerae*, adherence of *E. coli*, invasion of *E. coli* and *S. flexneri* and production and binding of toxin produced by *V. cholerae*. It was observed that none of the extracts were good or bad across all the bioassays. The spectral analysis showed multiple peaks correlating with a particular activity. Based on NMR and LC-MS/MS, it was noted that the extracts contained quercetin, ferulic acid and gallic acid. However, they did not correlate with the peaks that segregated extracts with good and poor activity.

**Conclusions:**

The current study identified key peaks in ^1^H NMR spectra contributing to the anti-diarrhoeal activity of guava leaf extracts. The approach of using spectral fingerprinting employed in the present study can thus be used as a prototype towards standardization of plant extracts with respect to efficacy.

**Supplementary Information:**

The online version contains supplementary material available at 10.1186/s12906-021-03221-5.

## Background

In recent years, phytomedicines have been gaining importance not only in developing countries like India and China but also in the developed countries like USA and Germany. Although their popularity amongst the population is rising, their acceptability by conventional medical practitioners is yet not satisfactory. One of the major reasons for this is the lack of data related to herbal medicine similar to that available for allopathic medicines, especially related to their standardization [1]. An additional factor for the lower acceptance of phytomedicines by the modern practitioners is the restricted acceptance of the holistic concept of herbal medicines which is contrary to the principle of single compound for single therapeutic activity in modern medicine [2].

This holistic approach of herbal medicines is often related to the synergistic action of its constituents. This makes standardization of extracts difficult and is therefore a neglected area leading to lack of uniform efficacy. The identification and quantification of a minimum of four phytoconstituents from a given extract meets the requirement for standardization for phytopharmaceuticals [3]. However, identification of biomarkers in the absence of knowledge of active principles may not be the most suitable approach towards standardization.

Metabolomics is fast becoming the approach of choice across broad fields of sciences as it gives insights into the chemical nature of biological material. In the field of medicinal plant research, the metabolomes of plants are a valuable resource for the evidence-based development of new phytotherapeutics and nutraceuticals. The most commonly used chromatographic techniques for metabolic profiling are Nuclear Magnetic Resonance (NMR) and Mass Spectroscopy (MS). Currently hyphenated techniques like Liquid Chromatography-Mass Spectroscopy (LC-MS) and Gas-Chromatography-Mass Spectroscopy (GC-MS) have also been used for their robustness and higher sensitivity of detection [1]. NMR-based studies due to its reproducibility have been undertaken for plant metabolites grown in varying controlled environments [4] or linkage of one or two activities to the metabolic profile of the extracts [5, 6]. Another application of this has been in quality control [7].

The current study used ^1^H NMR metabolic profiling and its correlation to anti-diarrhoeal activity of *Psidium guajava* (guava) leaf extracts, in an attempt to develop a prototype for standardization of extracts. The study was designed to demonstrate the utility of a spectral fingerprint without relying on identification of the active/marker compounds. The choice of guava leaves was on the basis of our previous work which established its anti-diarrheal activity [8]. Leaves of guava have been used as a traditional remedy for gastrointestinal disorders and are rich in phytoconstituents. The presence of several compounds such as sesquiterpene, saponins, sterols, triterpenoid, phenolics, coumarins, alkaloids and carotenoids has been reported in guava leaves [9–11]. Phenolics especially flavonoids are abundantly present in the leaves. Common polyphenolics found in guava leaves are protocatechuic acid, caffeic acid, gallic acid, ferulic acid and quercetin [12–14]. Though different types of extracts contain diverse types of compounds in varying amounts, Arya et al., reported that ethanolic and hydroalcoholic extracts of guava leaves contain higher quantities of phytochemicals [15]. Quercetin, is the most abundant flavonoid reported to be present in the leaves [16]. Interestingly, quercetin has been documented to be one of the active phytoconstituents of guava leaves and is reported to possess different pharmacological properties including antidiarrhoeal action [17–19].

For the present study, guava leaves were collected in different seasons from different locations and their ^1^H NMR profile were acquired. Eight bioassays representing important stages in diarrhoeal pathogenesis were undertaken with each collection and the results were correlated with the ^1^H NMR profiles using mathematical models. This work is one of the few studies in metabolomics, which has attempted to correlate the activity of multiple bioassays to ^1^H NMR signals in plant extracts obtained from an open field environment.

## Methods

### Plant material, preparation of extract

Mature leaves of the *Sardar* variety of guava were used for the study. This variety is widely cultivated in the state of Maharashtra, India. Leaves were collected from farms following verbal permission from the owners. Ninety samples of guava leaves were collected from individual trees; 10 from each of the three regions (Shirwal = W, Rahata = R and Dapoli = Da) in over three seasons (B = May 2013; C=October 2013 and D = March 2014). The leaves were authenticated by Dr. P. Tetali, an ethnobotanist. Individual voucher numbers were not obtained since this is a cultivated variety. However, a representative sample from one regional collection was deposited at the herbarium at Naoroji Godrej Centre for Plant Research (NGCPR, Shirwal, Maharashtra) under herbarium number NGCPR 712. All leaf samples were shade dried and powdered. A hydro-alcoholic (water and ethanol, 50:50) extract of each sample was prepared, lyophilized and the dried powdered extracts were then stored at − 80 °C. For all bioassays, extracts were reconstituted in distilled water and for ^1^H NMR in Deuterated water (D_2_O) and then used.

### Cell culture

HEp-2 (human laryngeal epithelial) cell line was procured from the National Centre for Cell Sciences, Pune, India. The cell line was routinely cultured in Dulbecco’s Modified Eagle’s Medium (DMEM, Gibco) supplemented with 10% fetal bovine serum (FBS, Bio-west), at 37 °C in a 5% CO_2_ atmosphere and maintained by passaging every 3–4 days.

### Bacterial strains used

Five bacterial strains a) enteropathogenic *Escherichia coli* (EPEC) strain B170, serotype 0111:NH (obtained from Centre for Disease Control, CDC, Atlanta); b) enterotoxigenic *E. coli*, heat labile toxin producer (ETEC) strain B831–2, serotype unknown (obtained from CDC); c) enteroinvasive *E. coli* (EIEC) strain EI34, serotype 0136:H- (kindly gifted by Dr. J. Nataro, Veterans Affairs Medical Centre, Maryland, USA); d) *Vibrio cholerae* Ogawa, serotype 01 (kindly gifted by Dr. S. Calderwood, Massachusetts General Hospital, Boston, USA) and e) *Shigella flexneri* M9OT, serotype 5 (kindly gifted by Dr. P. Sansonetti, Institut Pasteur, France) were used.

### ^1^H nuclear magnetic resonance - based (NMR) spectroscopy

The ^1^H NMR spectra were acquired at National Facility for High-Field NMR at Tata Institute of Fundamental Research (TIFR), Mumbai. 10 mg of lyophilized hydro-alcoholic extract of guava leaf was dissolved in 600 μl of D_2_O containing 0.1 mM solution of 3-(Trimethylsilyl)-propionic acid sodium salt (TSP, Sigma Aldrich) as reference. The samples were vortexed for 1 min, centrifuged and then transferred to a 5-mm NMR tube (Norell, NJ, USA). ^1^H NMR spectra were recorded for all the 90 samples. Spectra were also acquired for three compounds commonly found in guava leaves as standards. These were quercetin (purchased from Sigma), ferulic acid and gallic acid (both kindly gifted by Prof. KS. Laddha, Institute of Chemical Technology, Mumbai). 10 mg of the compound was dissolved in 600 μl D_2_O containing 0.1 mM TSP for spectral acquisition.

1D-NMR were recorded at room temperature (300 K) on Bruker Avance 800 MHz. Each spectrum was recorded for 256 scans using the noesygppr1d pulse sequence with an acquisition time 0.82 s, relaxation delay of 3 s and spectral width of 10,000 Hz. The FID (free induction decay) were zero-filled to 32 K data points for processing.

The raw data of the ^1^H NMR spectra acquired from the Bruker instrument was baseline corrected, phased and calibrated using the TOPSPIN Software (Version 3.6.0). The ^1^H NMR data post processing was imported into MATLAB software (Version R2014a) and all the spectra were aligned using a recursive segment-wise peak alignment method [20]. Regions showing signals for water were removed prior to multivariate statistical analysis. These spectra were exported from MATLAB in a text file format and bioassay results derived from the same samples were added to the dataset before importing into SIMCA (Version 14.1).

### Liquid chromatography–mass spectrometry (LC- MS/MS) analysis

To check the presence of quercetin, ferulic and gallic acid used as standards in ^1^H NMR, LC–MS/MS analysis was outsourced to Shobhaben Pratapbhai Patel School Of Pharmacy & Technology Management, SVKM’s Narsee Monjee Institute of Management Studies, Mumbai. The analysis was executed in a Shimadzu 8040 (Kyoto, Japan) instrument attached with a UV detector and a binary pump (G4220B). An electrospray ionization was the ionization source. Extract (five microliters, 1000 PPM in methanol) was injected into a Thermofisher C8 column (5 μm × 150 mm × 4.6 mm) with the oven temperature set to 30 °C. The mobile phase comprised of aqueous 0.1% formic acid (solvent A) and acetonitrile (solvent B) run with a gradient program. The standardized gradient method was set as time (min)/% B–0/5; 25/80; 28/80; 40/5; 45/5 with stop time at 45.0 min to ensure elution of all compounds. The flow-rate was set to 1 mL/min. The MS used was Triple Quadrupole in positive and negative ion modes. The optimized MS/MS parameters were: Gas temperature: 250 °C; Gas flow: 3 mL/min; nebulizer: 35 psi; MS range: 100–1000 m/z. LabSolutions® software was used for data acquisition. The name of phytocompound was assigned by comparing their mass and fragmentation patterns.

### Bioassays

All methods described below have been well established and validated earlier using suitable positive controls [8]. These included ofloxacin, lactulose and gallic acid for antibacterial activity, effect on bacterial colonization and effect on bacterial toxins respectively.

### Determination of concentration of extract used and establishing cut off limits

For each assay, pilot experiments were undertaken to determine an appropriate concentration which could give a wide spectrum of activity. All the extracts were then tested in the presence of the concentration of the guava extract finalized. Cut offs were set empirically to identify extracts with good (higher reduction/inhibition of the parameter) and poor (minimal reduction/inhibition of the parameter) activity. Values that were between the two cut offs were defined as intermediates.

The extract concentration used and the cut off values varied for each parameter and have been stated accordingly.

### Antibacterial activity

The antibacterial activity was carried out for *S. flexneri* and *V. cholerae* and determined by the agar dilution method [21]. The bacterial strains were plated on Mueller Hinton agar (Himedia laboratories, India) alone (as control) and at concentrations of 150 μg/ml and 600 μg/ml (wt/vol) of the reconstituted extract (as test) for *S. flexneri* and *V. cholerae* respectively. Two independent experiments were carried out. In each experiment, triplicate plates were used for control as well as the extract. Data was expressed as percent viability and number of colony forming units (cfu) in the control was taken as 100%.

### Effect on bacterial colonization

#### Effect on adherence

The effect on the adherence of *E. coli* B170 to HEp-2 cells was assayed as described previously [22]. Briefly, HEp-2 cells cultured on glass coverslips for 48 h were infected with a log phase culture (5 × 10^7^/ml) of the bacteria in DMEM in the absence of the extract (control) and presence of 20 μg/ml the extract (test) and incubated for 3 h. Non-adherent bacteria were washed off, the coverslips fixed and stained with toluidine blue stain (0.1% w/v). HEp-2 cells having typical EPEC microcolonies and/or > 5 adherent bacteria were counted under a light microscope. Two independent experiments were carried out. In each experiment, duplicate coverslips were set up for control and the extract. Data was expressed as percent adherence wherein the HEp-2 cells from the control was taken as 100%.

#### Effect on invasion

The effect on invasion of *E. coli* EI34 and *S. flexneri* to epithelial cells was studied as described elsewhere [23]. Briefly, a 24 h culture of HEp-2 cells grown in a 96-well tissue culture plate was infected with log phase culture (10^8^/ml) of the bacteria in DMEM alone (control) and in the presence of 20 μg/ml of the extract (test) and incubated for 2 h. Following incubation, the extracellular bacteria were washed off and the cells further incubated with DMEM containing gentamicin (100 μg/ml) for 2 h. Thereafter, the medium containing gentamicin was washed off and cells lysed with chilled distilled water. The released intracellular bacteria were enumerated by plating on nutrient agar. Two independent experiments were carried out. In each experiment, triplicate wells were set up for control and the extract. Data was expressed as percent viability and cfu from the control was taken as 100%.

### Effect on bacterial enterotoxins

#### Effect on *E. coli* heat labile toxin (LT) and cholera toxin (CT)

LT, which is localized in the cell membrane of *E. coli* B831–2 was obtained by lysing the bacterial cells with polymyxin B sulphate (1 mg/ml) whereas CT, which is released extracellularly, was obtained as a culture supernate of *V. cholerae*. To determine the effect of the extract on LT production, the *E. coli* cell lysate after polymyxin treatment following growth in absence of the extract (control) and in presence of 100 μg/ml extract (test) were used for the enzyme linked immunosorbent assay (ELISA). Similarly, for effect on CT production the culture supernatant of *V. cholerae* following growth in absence of the extract (control) and in presence of 75 μg/ml extract (test) served as the toxin in the ELISA as described below. The concentration of the extract to check the effect of the extract on the binding of the released CT was 50 μg/ml extract.

LT and CT were assayed by the ganglioside monosialic acid enzyme linked immunosorbent assay (GM1-ELISA) [24]. Briefly, the toxins (for production of LT/CT) were added to ELISA plates pre-coated with 1.5 μmol/ml of GM1. Anticholera toxin (1:300) and peroxidase labeled swine anti-rabbit immunoglobulin (1:200) were used as primary and secondary antibodies respectively. Orthophenylene diamine was used as the substrate. The intensity of the color thus developed was read at 492 nm in an ELISA plate reader. The protocol used for studying the effect of the extract on binding of CT was similar, except that the released CT was mixed with PBS (control) or the extract (test) before addition to the GM1 in the ELISA plate.

Two independent experiments were carried out. In each experiment, control and test wells were set up in triplicate. Data was expressed as percent toxin produced/bound and optical density of the control was taken as 100%.

### Presentation of data and statistical analysis

#### Bioassays

The data for each of the assay have been expressed as the Mean ± Standard deviation of the percentage values from two independent experiments. The percentage for each individual experiment was obtained using the formula {(C or T)/C} × 100; C being the mean value of the readings of control group and T being the mean value obtained in presence of the extract. Thus, the value of control is 100% and those of the test are percentages relative to control.

#### ^1^H NMR spectral data analysis

The spectral data were analysed using unsupervised Principal Component Analysis (PCA) and supervised Orthogonal Partial Least Square-Discriminant Analysis (OPLS-DA) in SIMCA (14.0).

Statistical link between the variation in biologic activity and the biochemical composition of the extracts was established using OPLS-Regression Analysis (OPLS-RA). Additional OPLS-DA analysis was carried out to observe the metabolic differences between the extracts with ‘good’ bioactivity and that with ‘poor’ bioactivity.

Apart from using different subsets of data for modeling, OPLS-RA was considered over the other models, since it considers actual values of bioassays. Failure of development of a significant model for certain bioassays using the supervised methods as well as the linear regression analysis led to exploration using Kruskal-Wallis test, a non- parametric model.

Further spectral signals correlating with the bioassay activities, were obtained from the S-plots of the significant models. Those signals that had correlation coefficient values above 0.6 were considered for establishment of the relationship between biological activity and ^1^H NMR profiles. Since SIMCA identified the different peaks based on the numerical values of the correlation coefficient there was a need to visualize the same in the graphical format. Hence MATLAB was used to overlap the spectra and each of the regions defined by the S-plot in SIMCA were visually checked to determine whether more than 60% of the spectra exhibiting either ‘good’/ ‘poor’ biological activity, used for the development of the model, showed greater height (intensity) with respect to the other spectra.

## Results

**Fig. 1:**
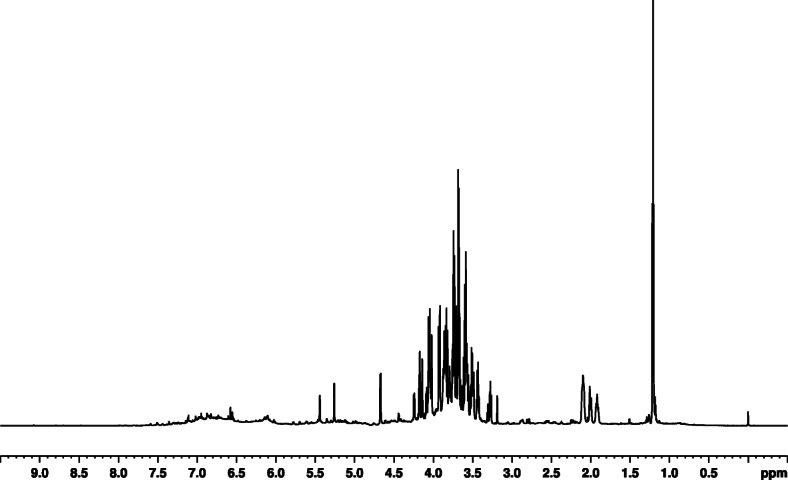
A representative ^1^H NMR spectrum of the guava leaf extract

A representative 1D ^1^H-NMR acquired for 90 guava extracts is depicted in Fig. 1 and representative ^1^H NMR spectra for seasonal and regional differentiation have been provided as Additional data (Supplementary Fig. S1 and Fig. S2 respectively). The ^1^H NMR spectra of quercetin, ferulic acid and gallic acid have also been given as Additional data (Supplementary Fig. S3, S4 and S5 respectively). Table 1 depicts the common ppm for standard compounds as compared to peaks in spectra of the representative guava leaf extracts presented as Additional data (Supplementary Figs. S1, S2, S3, S4, S5). As seen from this table, these three compounds were present. LC-MS/MS data also supported the presence of these three compounds (Table 2). The fragmentation patterns from LC-MS/MS have been provided as Additional data (Supplementary Figs. S6, S7, S8, S9, S10).

**Table 1:** Ppm values for standard compounds (Supplementary Figs. S3, S4 and S5) compared with peaks in spectra of representative guava leaf extracts (Supplementary Figs. S1 and S2)

		Individual guava leaf extract				
		WB	WC	WD	RD	DaD
**Compound**	**ppm**					
**Quercetin**	1.325	√	√	√		√
	1.334	√	√	√	√	√
	3.269	√	√	√	√	√
	3.565	√	√	√	√	√
	3.573	√	√	√	√	√
	3.63	√	√	√	√	√
	3.637	√	√	√	√	√
	3.643	√	√	√	√	√
	3.657	√	√	√	√	√
	3.663	√	√	√	√	√
	3.681	√	√	√	√	√
	3.772	√	√	√	√	√
	3.787	√	√	√	√	√
	3.919	√	√	√	√	√
	4.775					
	4.776					
	6.393	√				√
	6.413					
	6.937	√	√	√	√	√
	6.947	√	√	√	√	√
	7.15			√	√	√
	7.16	√	√		√	
	7.289	√			√	
**Ferulic acid**	3.903	√	√	√	√	√
	6.388	√	√	√	√	√
	6.408				√	√
	6.942	√	√	√	√	√
	6.952	√	√	√	√	√
	7.172	√	√	√	√	
	7.174	√		√	√	
	7.183	√	√		√	√
	7.185	√	√		√	√
	7.287	√	√	√	√	√
	7.289	√	√	√		√
	7.595	√	√			√
	7.615	√				
**Gallic acid**	3.885	√	√	√	√	√
	6.317			√		√
	6.336		√	√		√
	6.89	√	√	√	√	√
	6.907	√	√	√	√	√
	7.132	√	√	√	√	√
	7.575			√		√
	7.595	√	√		√	√

√ Present

WB, WC, WD - representatives for seasonal differentiation

WD, RD, DaD - representatives for regional differentiation

W – Leaves collected from region Shirwal; R - Leaves collected from region Rahata; Da - Leaves collected from region Dapoli

B - collection season May 2013; C – collection season October 2013; D – collection season March 2014

**Table 2:** LC-MS/MS data for the representative guava extracts^a^

Extract	Compound	Presence	Retention time (mins)	Mass/charge M/z	Collision energy
WB	Quercetin	✓	14.257	303 (M + H)^+^	25
	Ferulic acid				
	Gallic acid	✓	8.28	170.70 (M + H)^+^	35
WC	Quercetin	✓	13.91	302.35 (M + H)^+^	35
	Ferulic acid	✓	6.4	195.20 (M + H)^+^	35
	Gallic acid	✓	8.28	170.55(M + H)^+^	15
WD	Quercetin	✓	14.15	302.95 (M + H)^+^	35
	Ferulic acid	✓	6.4	195.15 (M + H)^+^	15
	Gallic acid				
RD	Quercetin	✓	14.15	303.10 (M + H)^+^	35
	Ferulic acid	✓	6.4	195.05 (M + H)^+^	25
	Gallic acid	✓	8.28	170.10 (M + H)^+^	15
DaD	Quercetin	✓	14.257	302.70 (M + H)^+^	35
	Ferulic acid				
	Gallic acid	✓	8.28	170.10 (M + H)^+^	35

Key:

✓ Present

WB, WC, WD - representatives for seasonal differentiation

WD, RD, DaD - representatives for regional differentiation

W – Leaves collected from region Shirwal; R - Leaves collected from region Rahata; Da - Leaves collected from region Dapoli

B - collection season May 2013; C – collection season October 2013; D – collection season March 2014

^a^The fragmentation patterns have been provided as Supplementary data

### Environmental influence

#### Seasonal differentiation

**Fig. 2:**
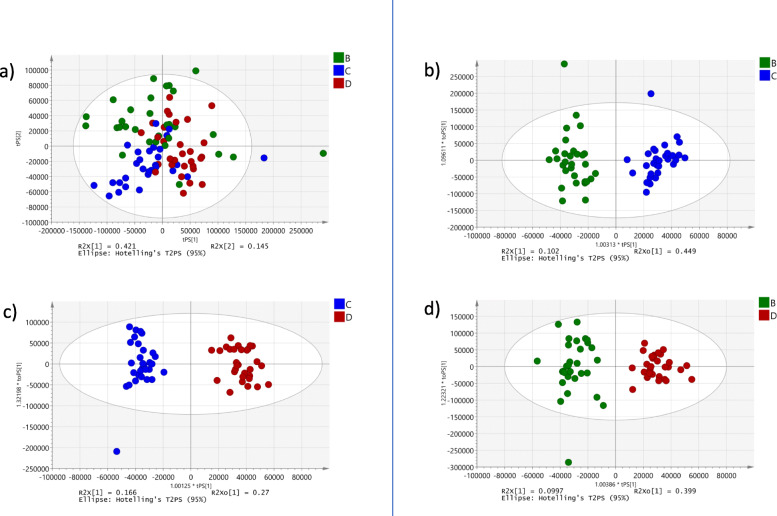
PCA plots for seasonal differentiation. **a**) PCA plot for seasonal data; **b**) OPLS-DA plot for season B and C; **c**) OPLS-DA plot for season C and D; **d**) OPLS-DA plot for season B and D. B: collection season May 2013; C: collection season October 2013; D: collection season March 2014

The PCA scores plot (Fig. 2) showed clear clustering based on the three seasons in which the samples were collected. The biggest variation between months May (B) and October (C), and March (D) was observed along the first principal component (PC1), whereas samples from May were separated from the other two seasons along the PC2. The pair-wise comparison were carried out using OPLS-DA analysis and statistically significant models were observed in May vs. October (R^2^X = 0.10; Q2(cum) = 0.79; *p* = 1.5 × 10^− 013^; Fig. 2b), October vs. March (R^2^X = 0.17; Q2(cum) = 0.748; *p* = 1.64 × 10^− 14^; Fig. 2c) and May vs March (R^2^X = 0.1; Q2(cum) = 0.77; *p* = 4.21 × 10^− 14^; Fig. 2d).

#### Regional differentiation

**Fig. 3:**
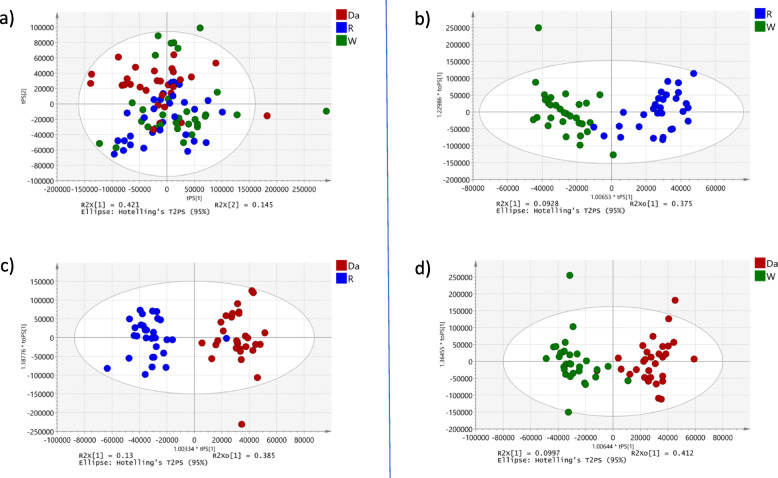
PCA plots for regional differentiation. **a**) PCA plot for regional data; **b**) OPLS-DA plot for W and R; **c**) OPLS-DA plot for R and Da; **d**) OPLS-DA plot for W and Da. W: Leaves collected from region Shirwal; R: Leaves collected from region Rahata; Da: Leaves collected from region Dapoli

The PCA scores plot (Fig. 3) showed clear clustering based on the three regions from which the samples were collected. The biggest variation between regions Rahata (R) and Dapoli (Da), and Shirwal (W) was seen along the first principal component (PC1), whereas samples from Dapoli (Da) were separated from other two regions along PC2. The pair-wise comparison were carried out using OPLS-DA analysis and statistically significant models were observed in Shirwal vs Rahata (R^2^X = 0.09; Q2(cum) = 0.552; *p* = 8 × 10^− 7^; Fig. 3b), Rahata vs Dapoli (R^2^X = 0.13; Q2(cum) = 0.757; *p* = 3.32 × 10^− 13^; Fig. 3c) and Shirwal vs Dapoli (R^2^X = 0.1; Q2(cum) = 0.68; *p* = 2.75 × 10^− 10^; Fig. 3d).

The differences observed between the samples from different seasons and regions could be the effect of changes in the environmental conditions such as precipitation, intensity of sunlight and temperature. Similar results have been shown earlier by others [25, 26].

### Correlation of ^1^H NMR spectral data with the biological activity

Correlation of the biological activity results (presented as Additional file Tables S1, S2, S3, S4, S5, S6, S7, S8) with the metabolomic profile of the extracts was undertaken.

The ppm values responsible for the significant differences, as identified from the models established for each bioassay is discussed below. Table 3 consolidates the ppm values of the prominent signals identified for individual bioassays.

**Table 3:** Ppm Intensity of the prominent signals identified for individual bioassays

A	B	C	D	E	F	G
**Peak ppm**						
0.738	3.436	1.125	**1.984**	**1.93**	**1.899**	**4.986**
0.8023	3.439	1.214	**1.988**	**4.04**	**1.917**	**4.909**
0.8358	3.514	3.663	**1.991**	**4.422**	**1.929**	
0.8623	3.728	4.017	**2.002**	**4.427**	**1.984**	
0.8778	3.735	4.019	**2.006**	**4.432**	**1.988**	
0.9048	4.664	4.021	**2.01**	**5.179**	**1.991**	
0.9288	4.673	4.129	**2.076**	**5.417**	**2.003**	
1.041		4.135	**2.08**	**5.483**	**2.006**	
1.049		4.139	**2.089**	**5.623**	**2.01**	
1.236		4.141	**2.094**	**5.632**	**2.076**	
1.395		5.24	**2.098**	**5.668**	**2.08**	
1.428		5.259	**2.105**	**5.692**	**2.208**	
1.448			**2.108**	**5.699**	**2.217**	
1.467			**2.111**	**6.819**	**2.23**	
1.481			**2.778**		**2.778**	
1.52			**2.785**		**2.785**	
1.543			**2.8**		**2.8**	
1.552			**2.807**		**2.807**	
1.572			**3.572**		**2.975**	
1.58			**4.03**		**4.985**	
1.586			**4.036**		**5.609**	
1.615			**4.042**		**5.622**	
1.67			**4.167**		**5.633**	
1.682			**4.171**			
1.711			**4.175**			
1.727						
1.742						
1.772						
1.797						
**1.898**						
**1.988**						
**2.006**						
**2.076**						
**2.094**						
**2.105**						
**2.109**						
**2.111**						
**2.23**						
**2.778**						
**2.785**						
**2.8**						
**2.807**						
**2.975**						
3.342						
3.353						
3.359						
3.536						
**4.171**						
**4.175**						
5.863						
5.872						
5.905						

A-Antibacterial activity against *S. flexneri*; B-Antibacterial activity against *V. cholerae*;

C- Adherence of EPEC; D- Invasion of EIEC; E- Invasion of *S. flexneri*; F- CT production; G-CT binding

Ppm values in bold represent peaks with prominence in extracts with good activity

### Antibacterial activity against *S. flexneri*

The antibacterial activity of the extract ranged from 0 to 137% as compared to the control. Cell viability ≤40% or ≥  90% were considered as effective or absence of inhibition of the growth. Based on this criterion, 21 extracts were regarded as good, while 58 extracts were poor and remaining 11 extracts were intermediates (Additional data - Table S1).

**Fig. 4:**
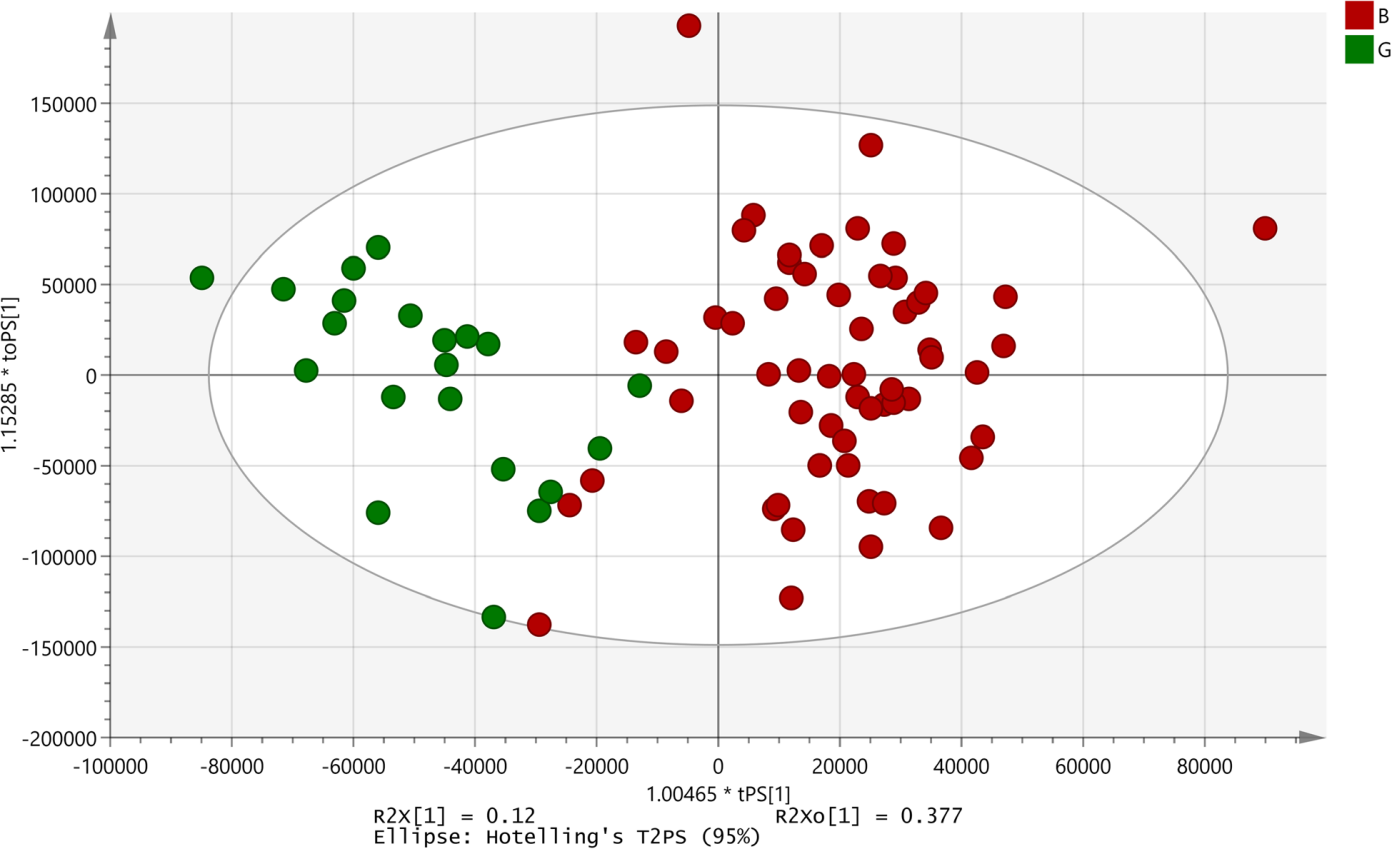
OPLS-RA model developed for antibacterial activity against *Shigella flexneri*. B: Poor activity; G: Good activity

The relationship between the metabolites and the antibacterial activity of the extract against *S. flexneri* was attempted using the regression model. The inclusion of all 90 samples did not develop a significant model. A subsequent OPLS model was established based on the samples exhibiting high and low inhibition. This model including 21 good activity extracts and 58 poor activity extracts showed a significant *p*-value of 4.73 × 10^− 10^; R^2^X = 0.12; Q2 (cum) = 0.561 (Fig. 4). A total of 63 signals had absolute correlation coefficient values greater than 0.6 in the S-plot, as being responsible for the differentiation between the high and low activity samples. Spectra of all the samples used for the development of the model were overlapped. Thirty six of the 63 signals identified by the S-plot, correlated with poor activity samples while 16 signals belonged to samples with good activity (Table 3).

### Antibacterial activity against *E. coli*

*E. coli* was highly resistant and even at an extract concentration of 1 mg/ml the bacterial growth was only partially inhibited (data not shown). Hence in the absence of marked inhibitory effect, further antibacterial assays and the subsequent metabolomic data analysis with *E. coli* was not undertaken.

### Antibacterial activity against *Vibrio cholerae*

The antibacterial activity of the extract ranged from 7 to 138% as compared to the control. For this assay, percent viability ≤40% was considered as greater inhibition of growth/ ‘good activity’ and percent values ≥80% was taken as absence of inhibition/ ‘poor activity’. Thus seven extracts were good, 45 extracts were poor and the remaining 38 extracts were intermediates (Additional data - Table S2).

**Fig. 5:**
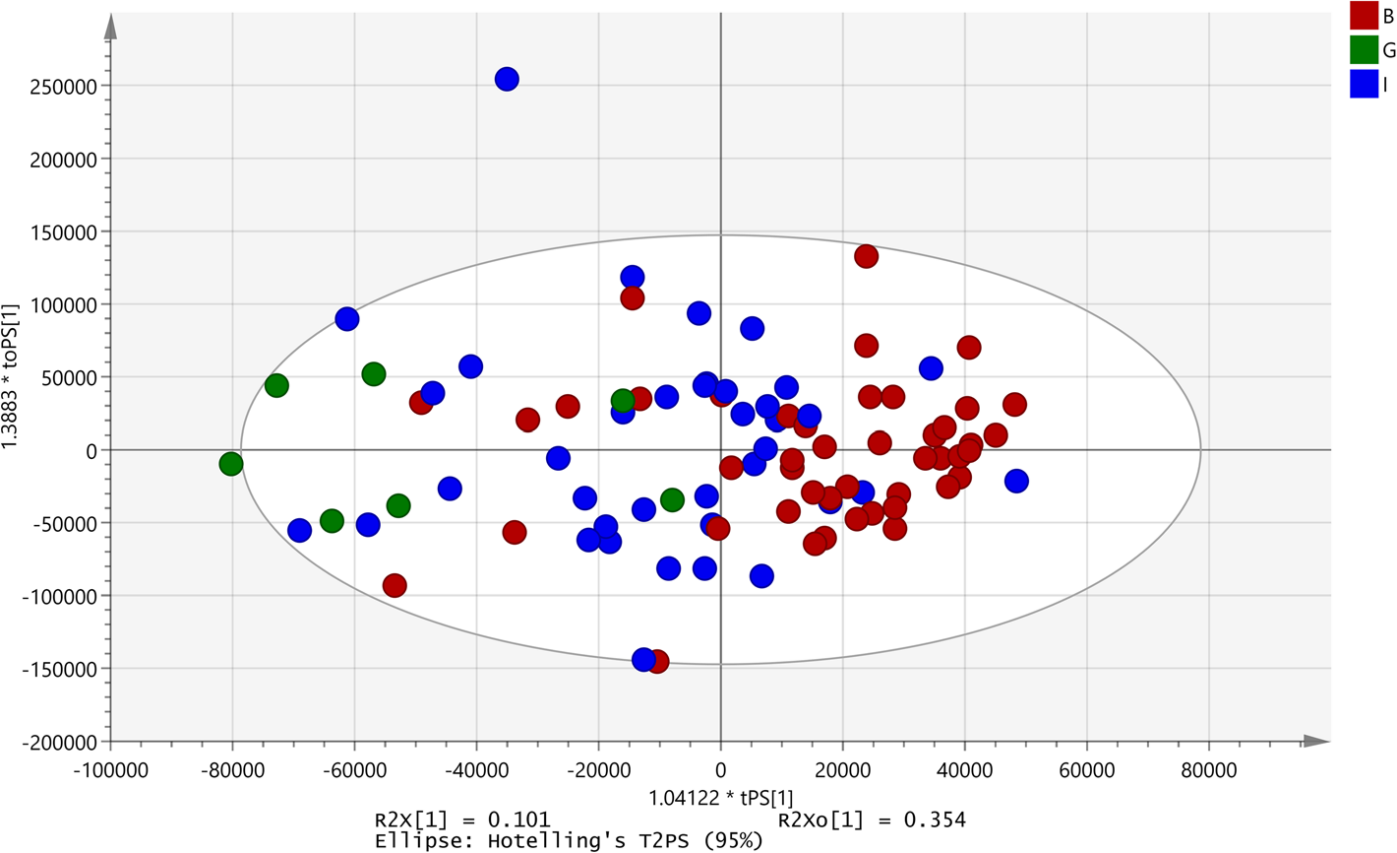
OPLS-RA model developed for antibacterial activity against *Vibrio cholerae*. B: Poor activity; G: Good activity; I: Intermediate activity

The correlation between antibacterial activity of the extract against *V. cholerae* and the ^1^H NMR profiles was established using OPLS regression analysis (*p*-value of 0.03; R^2^X = 0.101; Q2 (cum) = 0.151; Fig. 5). The S-plot of this model identified 10 signals that differentiated the extracts based on their activity. Out of the 10 signals identified by the S-plot, in seven the intensity of the signals was higher in spectra of samples with poor activity and remaining 3 signals showed a mix trend (Table 3).

### Effect on bacterial adherence

It was noted that the percent bacterial adherence in presence of the extract ranged from 0 to 99% as compared to the control. For this assay, bacterial adherence ≤30% was taken as good inhibitory activity and ≥ 60% was taken as an indicator of poor activity. Hence 25 extracts had good activity, 19 extracts had poor activity and 46 extracts had intermediate activity (Additional data - Table S3).

**Fig. 6:**
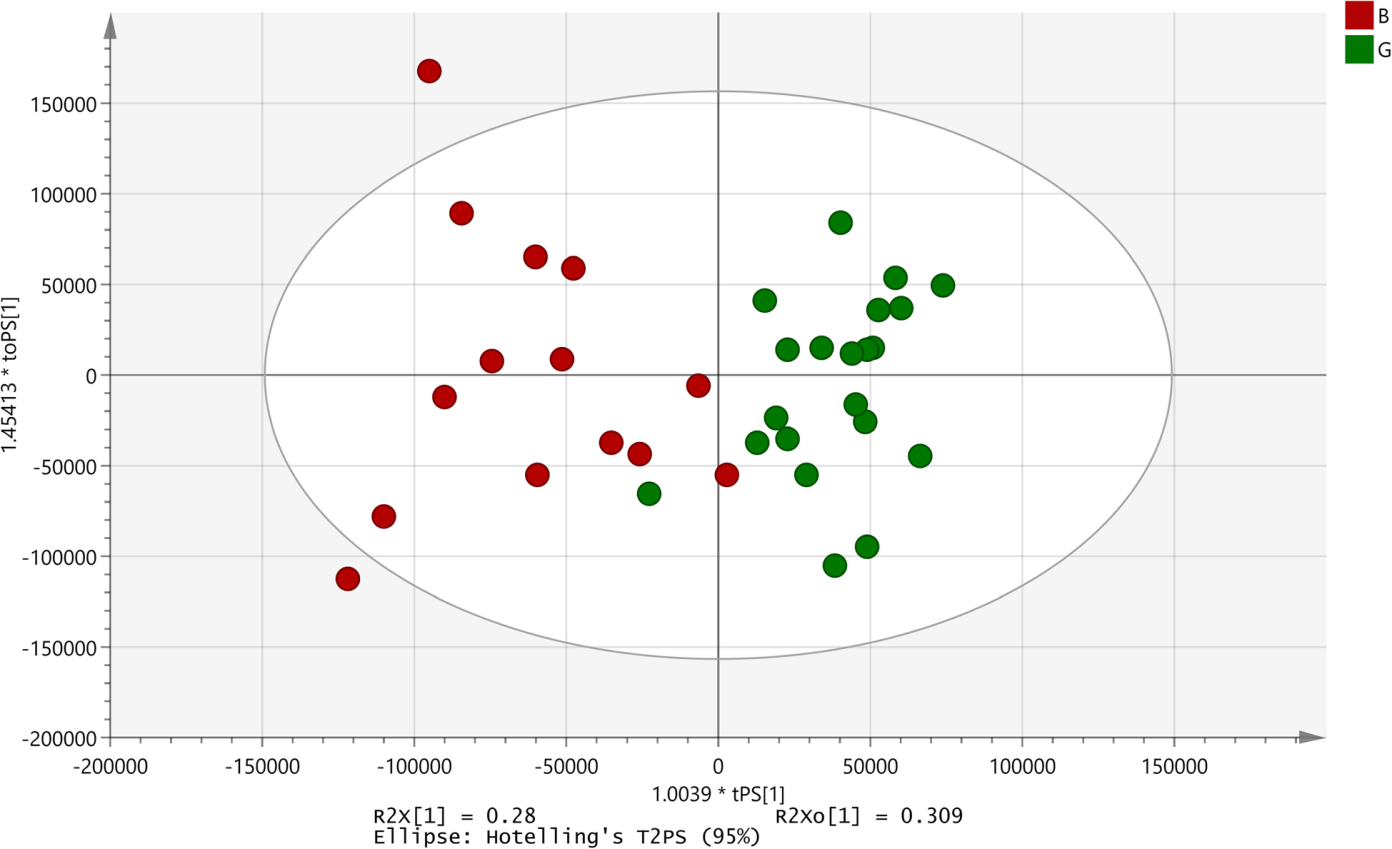
OPLS-DA model developed for effect on adherence of EPEC to HEp-2 cells. B: Poor activity; G: Good activity

A significant OPLS-DA model with a *p*-value of 0; R^2^X =  0.28; Q2(cum) =  0.564, was successfully developed by considering 21 good activity extracts and 14 poor activity samples after exclusion of confounders (4 good and 5 poor activity extracts, Fig. 6). In the S-plot, the model identified 39 signals that differentiated the samples based on activity; only 12 peaks correlating with poor activity could be identified (Table 3).

### Effect on bacterial invasion

#### EIEC

The percent bacterial invasion in presence of the extract ranged from 7 to 73% as compared to the control. For this assay, percent invasion ≤20% was taken as greater inhibition of bacterial invasion and percent values ≥40% was considered to be absence of inhibition. Hence 22 extracts were considered as good, 13 extracts were poor and 55 extracts had intermediate activity (Additional data - Table S4).

**Fig. 7:**
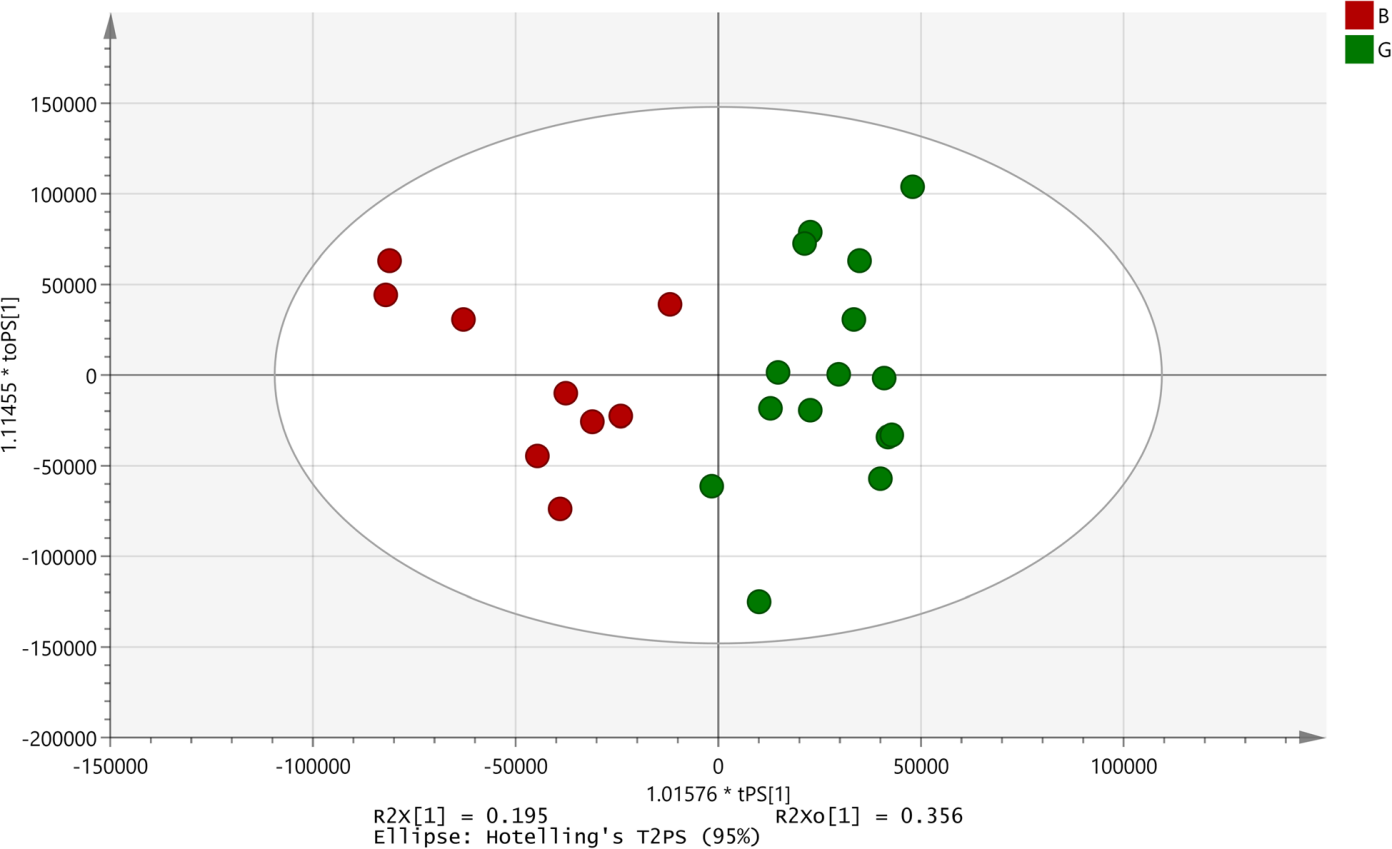
OPLS-DA model developed for effect on invasion of EIEC into HEp-2 cells. B: Poor activity; G: Good activity

Numerous attempts were made to derive a significant model. Models based on inclusion of all the samples, only the samples showing good and poor activity and even the regression analysis did not show significance. Hence based on the OPLS-DA plot derived from the good versus the poor activity model, certain borderline samples (those in the overlapping region between the good and the poor activity) were omitted (good-7; poor-4). The new set of samples (good-15; poor-9) thus derived were reanalysed and a significant OPLS-DA model with a *p*-value of 0 was established (Fig. 7). The other parameters of this model were R^2^X = 0.195 and Q2(cum) = 0.678. In the S plot, the model developed identified 65 signals responsible for differentiation out of which 25 signals showed higher intensities in samples with good activity. The remaining could not be assigned to any group since they were equally present in both (Table 3).

#### S. flexneri

The percent bacterial invasion in presence of the extract ranged from 2 to 140% as compared to the control. For this assay, percent invasion ≤20% was taken as greater inhibition of bacterial invasion and percent values ≥40% was considered as absence of inhibition. Thus, 28 extracts had good activity, 19 extracts had poor activity and remaining 43 were intermediates (Additional data - Table S5).

**Fig. 8:**
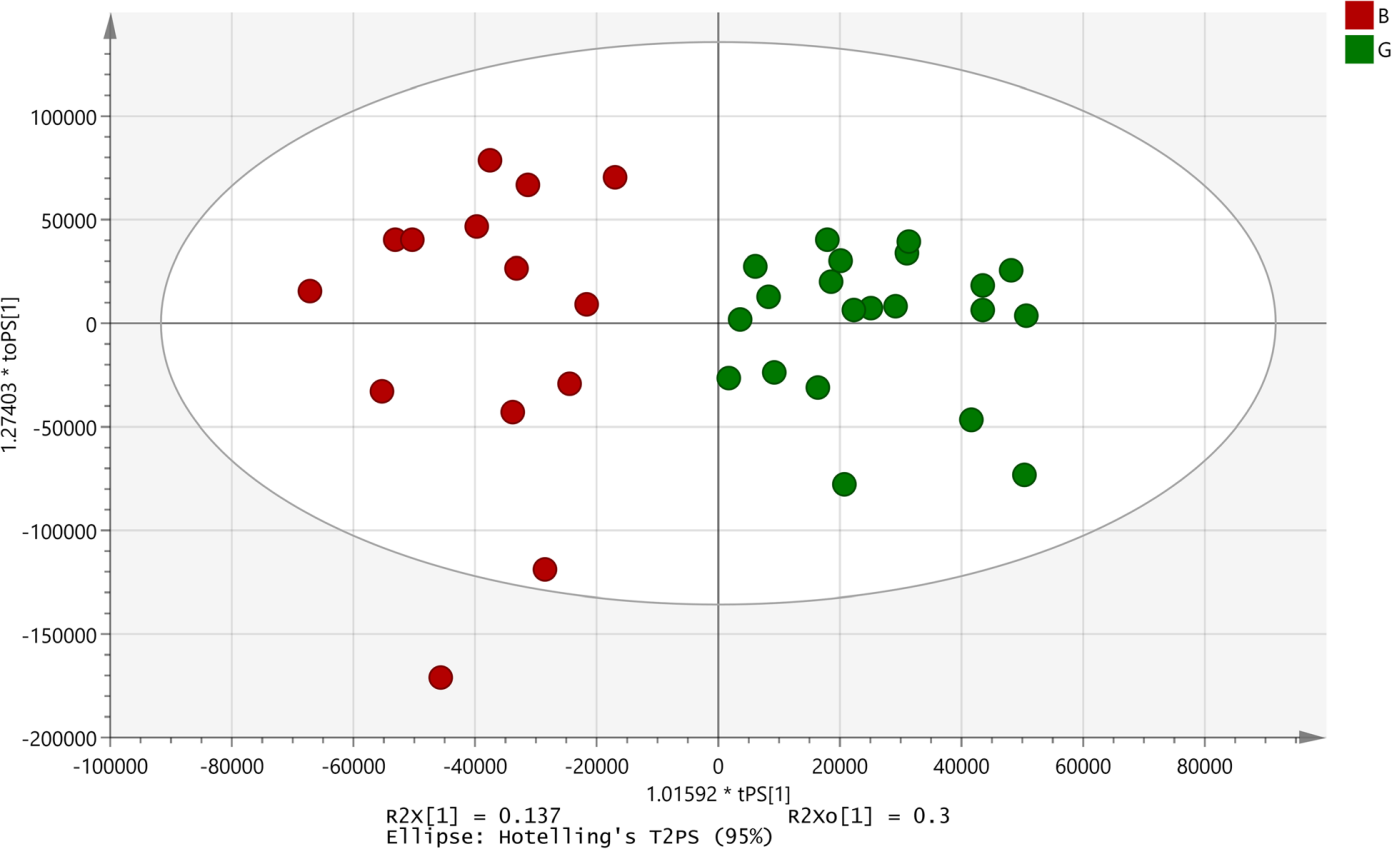
OPLS-DA model developed for effect on invasion of *Shigella flexneri* into HEp-2 cells. B: Poor activity; G: Good activity

A similar approach as that applied to analyse the data for invasion by *E. coli* into HEp-2 cell lines was applied to this activity data set. The number of omitted samples were 12 (good-7; poor-5) and a significant OPLS-DA model was generated with 35 samples (good-21; poor-14). A *p*-value of 0, R^2^X = 0.137 and Q2 (cum) = 0.507, was established (Fig. 8). The model identified 75 signals as those responsible for the differentiation out of which in 14 signals, samples with good activity showed higher intensities (Table 3). None of the signals correlated with samples with poor activity. The remaining peaks comprised of a mixture of good and poor activity samples.

1

#### Effect on CT production

The toxin production in presence of the extract, ranged from 30 to 83% as compared to the control. For this assay, values ≤40% was considered as greater inhibition in toxin production and values ≥60% was taken as absence of inhibition. Thus 35 extracts were good, 22 extracts were poor and 33 had intermediate activity (Additional data - Table S6).

**Fig. 9:**
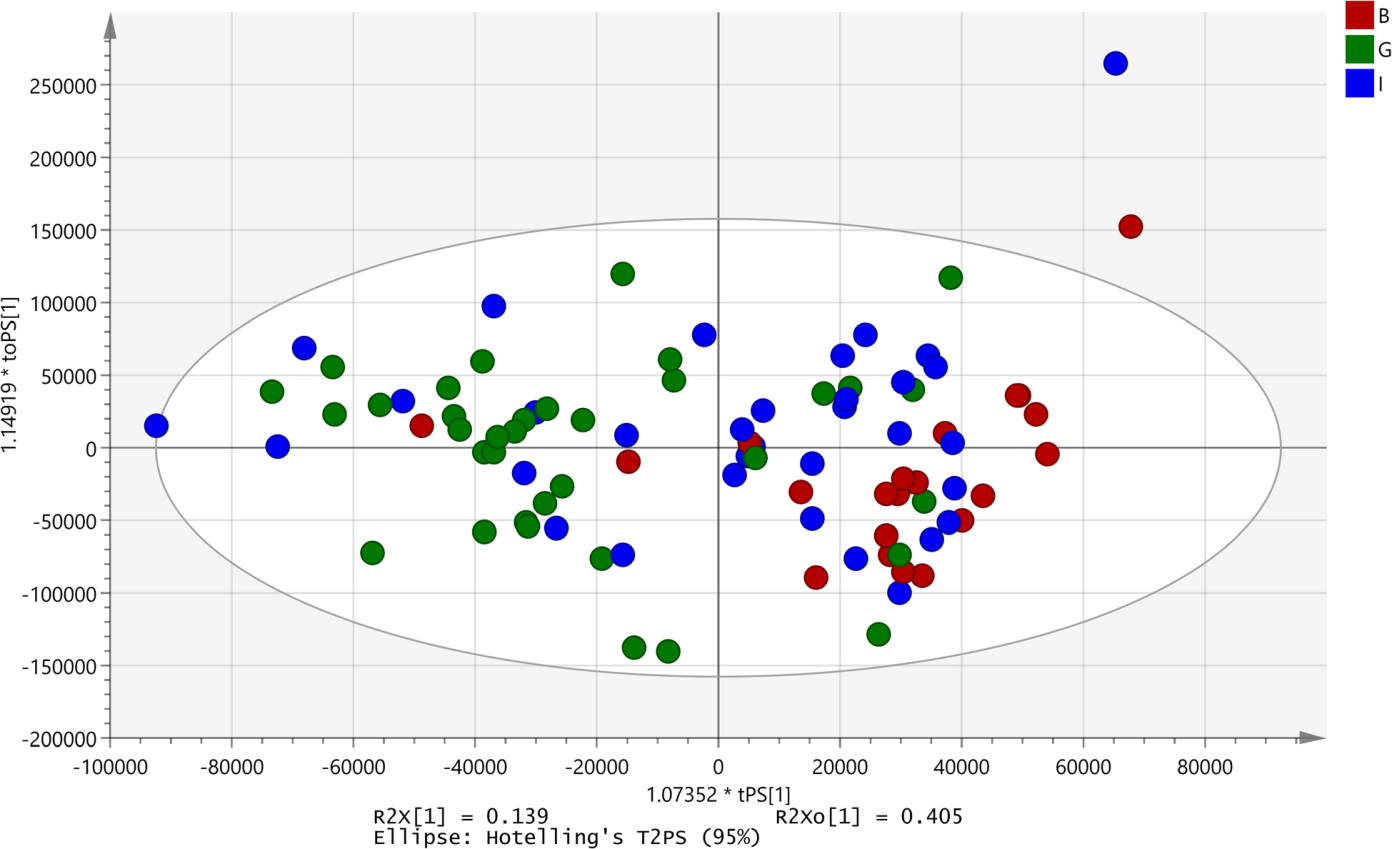
Regression model developed for production of cholera toxin by *Vibrio cholerae*. B: Poor activity; G: Good activity; I: Intermediate activity

A significant regression model with a *p*-value of 0, R^2^X = 0.139 and Q2 (cum) = 0.183, was developed using all 90 samples (Fig. 9). Out of the 52 signals identified by the S-plot as those responsible for differentiation, only 23 signals could be assigned to extracts with good activity. The remaining peaks comprised of extracts with both good and poor activity (Table 3).

#### Effect on CT binding

The toxin binding in presence of the extract, ranged from 27 to 72% as compared to the control. For this assay, values ≤40% was taken as indicator of greater reduction in toxin binding and values ≥50% was considered as absence of activity. Thus 42 extracts had good activity, 27 extracts had poor activity and 21 were in the intermediate range (Additional data - Table S7).

**Fig. 10:**
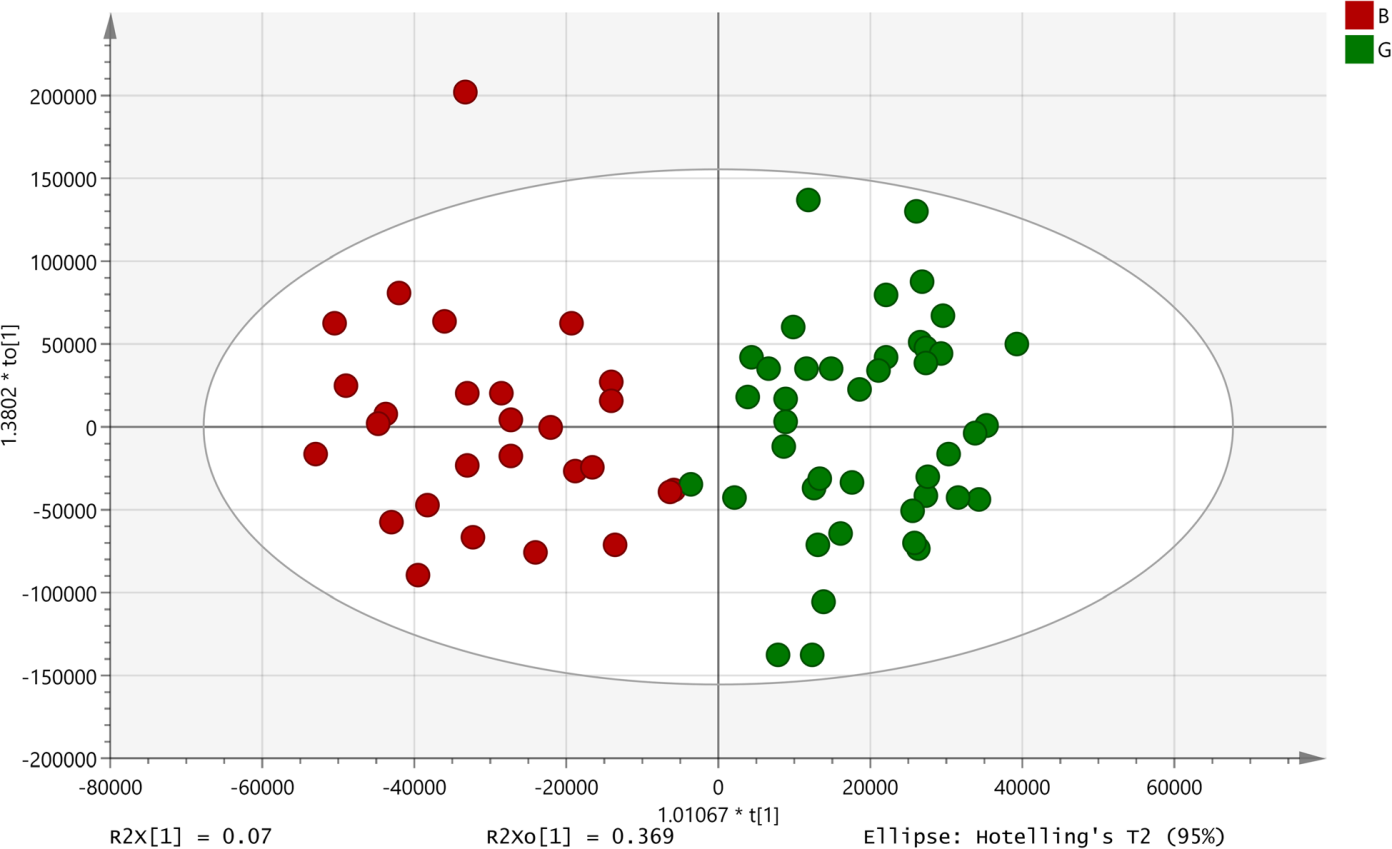
OPLS-DA model developed for binding of cholera toxin to GM1 receptor. B: Poor activity; G: Good activity

An OPLS-DA model with a significant *p*-value of 1.15 × 10^− 6^, R^2^X = 0.07 and Q2(cum) = 0.53, including 42 extracts with good activity and 27 with poor activity was developed (Fig. 10). Only two signals got identified as those responsible for the differentiation and in both the extracts with good activity showed a higher intensity (Table 3).

#### Effect on LT production

The toxin production in presence of 100 μg/ml extract, ranged from 27 to 102% as compared to the control (toxin production in media alone was considered as 100%). Values ≤40% indicate greater reduction in toxin production and values ≥60% were considered to have no effect on toxin production. Thus 22 extracts had good activity, 35 extracts had poor activity and 33 extracts were in the intermediate range (Additional data - Table S8).

All methods listed earlier were tried but a successful model could not be developed.

## Discussion

The aim of this study was to develop a prototype for standardization of crude extracts using the anti-diarrhoeal activity of guava leaves as an example. Diarrhoea was selected due to the high mortality rates, increased incidence of antibiotic resistance and its widespread prevalence in developing countries. Diarrhoea is the eighth leading cause of mortality accounting for over 1.6 million deaths [27]. Increased antibiotic resistance in diarrhoeal pathogens has been reported [28–32] necessitating the development of alternatives. It is envisaged that medicinal plants can fulfil this niche.

Plant extracts are complex mixtures and contain a number of compounds. Some of these may be present in small amounts but nevertheless contribute to efficacy which often may be through some synergistic action with other constituents [33]. Hence as a holistic approach, the total profiling of an extract in the form of a representative fingerprint is required. Fingerprint analysis or characteristic profiling has been accepted by World Health Organization (WHO) and other organizations (Food and Drug Administration, FDA; State Food and Drug Administration of China, SFDA and European Medicines Agency, EMA) as a methodology for the quality control of herbal samples [34–37]. Fingerprinting is considered distinctive and forms a benchmark for a particular extract especially when the identity of the active principle(s) is unknown [38]. Hence it is in tune with the concept that the entire plant could represent a drug itself and not just a single compound [39]. In high resolution spectra such as ^1^H NMR used in the present study, fingerprinting ignores the assignment problem arising due to the multitude of signals and on the contrary, through multivariate analysis compares set of spectra and thus the samples from which the spectra were obtained [40]. The current study aimed at the spectral fingerprint profile of guava leaf extract for characterization of extracts showing antidiarrhoeal activity.

Guava leaves have been used globally in the treatment of gastrointestinal disorders [41, 42]. Our work which included a clinical trial has confirmed that guava is a promising anti-diarrhoeal plant exhibiting a wide spectrum of activity [8, 43–46]. However, its functional components largely remained unknown, mainly due to chemical complexity and possible synergism.

The hydroalcoholic extract of guava leaves have been reported to have several phytoconstituents that include flavonoids, tannins, triterpenoids, saponins, sterols, alkaloids and carbohydrates [15, 47]. All these constituents have varied pharmacological actions. Phenolic acids such as gallic and ferulic acid are reported to possess antimicrobial, anti-carcinogenic, anti-inflammatory, hepatoprotective activities [48]. Similarly, anti-inflammatory and anti-oxidant activities are also associated with ferulic acid, catechins and quercetin [49, 50]. Flavonoids play a significant role in scavenging different reactive oxygen species [51]. Ellagic acid also has several pharmacological activities such as antioxidant, antimicrobial, anti-inflammatory and antiestrogenic [52]. Due to the presence of these compounds, guava leaves have been attributed with several biological activities.

Besides the above pharmacological properties, the anti-diarrhoeal activity of the compounds has also been documented. Chen et al., [53] have reported that gallic acid can prevent binding of *E. coli* toxin to ganglioside receptor. Presence of gallic acid has also been associated with antidiarrhoeal action due to its anti-secretory properties [54]. Quercetin is known to have antispasmolytic activity [17]. Protective effect of ellagic acid in castor oil and magnesium sulfate induced diarrhoea in mice has also been studied [52, 55]. The antispasmodic activity of saponins using guinea pig ileum has been demonstrated [56]. Other flavonoids such as rutin, kaempferol and morin have been reported to reduce the intestinal transit time in the mouse model [57]. Rutin is also said to have antidiarrhoeal properties as it prevents entry of rotavirus and/or viral interactions with target cells in the gut [58]. Studies by Yi et al., [59] on senna induced diarrhoea in mice showed that presence of flavonoids like rutin, isoquercitrin, quercitrin, myricetin exhibit anti-diarrhoeal properties by controlling the abnormal increase in vascular permeability and alleviating the inflammatory response. Epicatechin has been shown to prevent cholera toxin induced diarrhoea [60].

It is possible that different constituents of the guava leaf extract would be responsible for individual activities. Hence multiple compounds in the whole extract could act against different parameters resulting in an additive effect which would lead to a wider spectrum of antidiarrhoeal activity. Alternatively, some compounds my act as inhibitors and mask the activity of some other compounds.

Besides this additive feature, synergism could also play a role. In studies related to infectious diarrhoea caused by *S. flexneri*, it has been shown that quercetin alone is not responsible for the action; rather the efficacy of the whole extract is due to the synergistic effect of two or more phytoconstituents [8, 61]. This has also been exemplified by the observation that bio-guided fractionation often results in loss of activity [62]. In the present study, the ^1^H NMR spectra also highlighted this, for example 14 peaks were identified to be responsible for enhanced inhibition of invasion by *S. flexneri* into HEp-2 cells (Table 3).

Standardization of herbal extracts remains a challenge and appropriate methods are required. These methods would then ensure proper quality control, reproducibility and accountability of the materials along with the establishment of efficacy and safety profiles [1].

Profiling of the extracts using different analytical techniques, so as to decipher the components is a multistep process due to the large number of metabolites present and varied chemical nature of the individual components [1]. Identification of metabolites is not only challenging but also time consuming [63] necessitating development of high through put methods. This has led to the development of metabolomics towards a detailed, holistic and systemic analysis of all metabolites present [64]. Various techniques such as NMR, FTIR, MS, LC-MS, GC-MS have been used. As the amount of data obtained through these platforms is often large, it demands the use of multivariate statistics [64]. This combination has emerged as a promising tool towards detailed evaluation of metabolite data especially in case of complex/multicomponent mixtures. In targeted metabolomics, specific metabolites are profiled with known marker compounds not necessarily related to bioactivity [65]. The data set obtained using the target approach is simple and permits quantification of the desired metabolite(s) [66]. On the other hand, untargeted metabolomics profiles all possible metabolites present and thus provides a fingerprint rather than identifying any particular metabolite(s) [67]. Thus, the untargeted metabolomics is not limited by the need to know which chemical components are indicators of efficacy.

Over the years, metabolomics is being increasingly employed in herbal medicine for applications such as assessing quality, detecting adulterants, evaluating biological efficacy and determining optimum conditions for cultivation [68, 69]. A review by Ning et al., [70] comprehensively describes the various applications of metabolomics citing the examples from traditional Chinese Medicine.

However, there is a dearth of reports on the use of non-targeted metabolomic fingerprinting as a standardization technique. Standardization of *Ayurvedic* polyherbal formulations *Triphala* and *Trikatu* used targeted metabolite profiling [71, 72]. Another polyherbal formulation by trade name ‘Linkus’ containing *Adhatoda vasica* as an ingredient was standardized by a combination of untargeted and targeted metabolomics using chemometric as well as regression analysis of *A. vasica* collected from different locations [73].

The present work differs from the existing studies in the field of metabolomics. It used untargeted metabolomics using ^1^H NMR along with multivariate analysis for guava leaf extracts collected over different seasons and different locations. The aim was to correlate the spectral data with anti-diarrhoeal activity of guava leaves using representative bioassays. It also employed multiple assays to arrive at the identification of fingerprints representing efficacious extracts and aid differentiation of good vs poor batch of leaves. The study did not primarily intend to recognize any singular biomarker(s) correlating with the anti-diarrhoeal activity of guava leaves, rather it aimed at identifying a spectral fingerprint comprising of the desired peaks. The importance of fingerprinting has also been stressed by other workers involved in the study of traditional Chinese medicine [74, 75].

Thus NMR was the method of choice since the results are easily reproducible and sample preparation minimal thus it is more frequently used. Although ^1^H NMR profiling has lower sensitivity and resolution power it is preferred over MS due to its other advantages. ^1^H NMR profiling with its minimal and non-destructive sample preparation has higher reproducibility with quantitation being possible without the availability of a standard. It can be used for all types of compounds including non-ionizable compounds which cannot be detected by MS [1]. An added advantage of ^1^H NMR spectroscopy is that it helps in identifying both the primary and secondary metabolites simultaneously without the need for any kind of fractionation/separation. The primary metabolites usually are represented by signals in the aliphatic region (0–4.6 ppm) while the secondary metabolites are found in the aromatic region (5-11 ppm) [76]. Thus, ^1^H NMR profiles of the extracts indicate all the possible signals due to presence of ^1^H in the metabolites irrespective of the matrix in which they are present. Despite a single peak not necessarily representing a single compound and often multiple peaks being the signature of a single compound which is also reflected in the ^1^H NMR spectra acquired for the standard compounds (Supplementary Figs. S3, S4 and S5), ^1^H NMR spectroscopy was chosen for the current study because of its advantages.

Statistical analysis comprising of supervised and unsupervised methods (PCA and OPLS-DA) were used to establish the relationship between the different signals in the ^1^H NMR spectra of the extracts and the biological activity associated with that extract. Since the samples were collected from open field conditions, there was a high degree of variation. Hence statistical models could not sometimes be successfully developed considering the data from all the extracts and a subset was used to develop a significant relationship.

Firstly, the analysis tried to correlate the results of a single bioassay with the signals from the spectra. The results showed that multiple signals were identified correlating with individual activity. Some of these signals were predominantly seen in good activity extracts while some signals correlated with samples having poor activity. However, some signals did not show predominance in either of the good or poor activity extracts despite contributing to the differentiation in the model developed. This was assumed to be due to the limitations associated with the statistical models used and also the large variation in the metabolite profile of the extracts due to the open environment the guava trees were exposed to.

The ^1^H NMR spectral data for the guava extracts showed signals in the aliphatic and aromatic regions, suggesting that both primary and secondary metabolites were present in these hydroalcoholic extracts. Though The OPLS-DA analysis could clearly distinguish between the different regions and seasons as also reported earlier by others [5–7], the results showed that no extract showed either good or poor activity across all seven bioassays undertaken. Even with respect to a single assay, multiple peaks were identified correlating with the activity (Table 3). Quercetin, ferulic acid and gallic acid were present in the extracts based on NMR (Table 1) and LC-MS/MS (Table 2). However, they did not correlate with the peaks that segregated extracts with good and poor activity and thus they could be only used as markers.

Moreover due to the large number of primary and secondary metabolites present in the extract there are overlapping signals which may not clearly allow identification of the individual components involved. Additionally, it was observed that some peaks correlated strongly with poor activity implying that they may be inhibitory constituents. Thus, it was not possible to identify compounds that were responsible for the anti-diarrhoeal activity as a whole or for the individual bioassays. Previous studies attempting to identify the compound(s) responsible for activity through fractionation found that the activity is often significantly reduced in the isolated fraction [62]. Another approach wherein presence of markers has been used for the standardization of extracts has also run into difficulty since while the marker compounds could be present, the active component(s) may or may not be present in a significant amount to show the desired efficacy. As seen from Table 3 a number of peaks were identified for a single bioactivity which suggests an interplay of compounds in achieving the final outcome. This justifies our approach of relying on a fingerprint consisting of the desired peaks and not identifying compound(s) thus bypassing the above limitations. The choice of NMR was therefore crucial due to its reproducibility and minimal sample preparation. Thus, the current study has demonstrated that it is possible to standardize extracts with respect to activity based on a fingerprint rather than relying on markers.

## Conclusion

With increase in use of traditional medicine, innovative methods and approaches towards their standardization are needed in phytomedicine research. The current study attempted to recognize key peaks from ^1^H NMR spectra which together would comprise of a spectral fingerprint relating to efficacy of guava leaf extract when a number of unidentified active principles are involved. This was met through the identification of key signals responsible for the differentiation of extracts with good and poor activity, establishing it as a prototype for standardization of a plant extract.

## Supplementary Information

**Additional file 1: Fig. S1.** Representative ^1^H NMR plots for seasonal differentiation. Individual plots are representative ^1^H NMR spectrum of the guava hydroalcoholic extract prepared from leaves collected from Shirwal (W region) in. a) season B (May 2013); b) season C (October 2013); c) season D (March 2014).

**Additional file 2: Fig. S2.** Representative ^1^H NMR plots for regional differentiation. Individual plots are representative ^1^H NMR spectrum of the guava hydroalcoholic extract prepared from leaves collected in season D (March 2014) from. a) Shirwal (W region); b) Rahata (R region); c) Dapoli (Da region).

**Additional file 3: Fig. S3.** ^1^H NMR plot acquired for quercetin.

**Additional file 4: Fig. S4.** ^1^H NMR plot acquired for ferulic acid.

**Additional file 5: Fig. S5.** ^1^H NMR plot acquired for gallic acid.

**Additional file 6: Fig. S6.** Fragmentation patterns for compounds identified by LC-MS/MS in extract WB. W: Leaves collected from Shirwal region; B: May 2013 collection.

**Additional file 7: Fig. S7.** Fragmentation patterns for compounds identified by LC-MS/MS in extract WC. W: Leaves collected from Shirwal region; C: October 2013 collection.

**Additional file 8: Fig. S8.** Fragmentation patterns for compounds identified by LC-MS/MS in extract WD W: Leaves collected from Shirwal region; D: March 2014 collection.

**Additional file 9: Fig. S9.** Fragmentation patterns for compounds identified by LC-MS/MS in extract RD. R: Leaves collected from Rahata region; D: March 2014 collection.

**Additional file 10: Fig. S10.** Fragmentation patterns for compounds identified by LC-MS/MS in extract DaD. Da: Leaves collected from Dapoli region; D: March 2014 collection.

**Additional file 11: Table S1.** Values represent Mean ± SD of % bacterial (*Shigella flexneri*) viability from two independent experiments done in triplicates. B: May 2013 collection; C: October 2013 collection; D: March 2014 collection. W: Shirwal; R: Rahata; Da: Dapoli

**Additional file 12: Table S2.** Values represent Mean ± SD of % bacterial (*Vibrio cholerae*) viability from two independent experiments done in triplicates. B: May 2013 collection; C: October 2013 collection; D: March 2014 collection. W: Shirwal; R: Rahata; Da: Dapoli

**Additional file 13: Table S3.** Values represent Mean ± SD of % bacterial (EPEC) adherence from two independent experiments done in duplicates. B: May 2013 collection; C: October 2013 collection; D: March 2014 collection. W: Shirwal; R: Rahata; Da: Dapoli

**Additional file 14: Table S4.** Values represent Mean ± SD of % bacterial (*E. coli*) invasion from two independent experiments done in duplicates. B: May 2013 collection; C: October 2013 collection; D: March 2014 collection. W: Shirwal; R: Rahata; Da: Dapoli

**Additional file 15: Table S5.** Values represent Mean ± SD of % bacterial (*S. flexneri*) invasion from two independent experiments done in duplicates. W: Shirwal; R: Rahata; Da: Dapol

**Additional file 16: Table S6.** Values represent Mean ± SD of % cholera toxin (CT) production from two independent experiments done in triplicates. B: May 2013 collection; C: October 2013 collection; D: March 2014 collection. W: Shirwal; R: Rahata; Da: Dapoli

**Additional file 17: Table S7.** Values represent Mean ± SD of % CT binding from two independent experiments done in triplicates. B: May 2013 collection; C: October 2013 collection; D: March 2014 collection. W: Shirwal; R: Rahata; Da: Dapoli

**Additional file 18: Table S8.** Values represent Mean ± SD of % labile toxin production from two independent experiments done in triplicates. B: May 2013 collection; C: October 2013 collection; D: March 2014 collection. W: Shirwal; R: Rahata; Da: Dapoli

## Data Availability

All the data has been either included in the manuscript or given as Additional file.

## Abbreviations

D_2_O: Deuterated water
EPEC: Enteropathogenic *Escherichia coli*
EIEC: Enteroinvasive *Escherichia coli*
ETEC: Enterotoxigenic *Escherichia coli*
LT: Labile toxin
CT: Cholera toxin
NMR: Nuclear Magnetic Resonance
LC-MS: Liquid Chromatography- Mass Spectroscopy
PCA: Principal Component Analysis
OPLS-DA/RA: Orthogonal Partial Least Square-Discriminant Analysis/Regression Analysis
GM1-ELISA: Ganglioside monosialic acid enzyme linked immunosorbent assay
