# Supplementary figures and images for: 1H nuclear magnetic resonance-based metabolite profiling of guava leaf extract: an attempt to develop a prototype for standardization of plant extracts
Source: BMC Complement Med Ther. 2021 Mar 18;21:95. doi: 10.1186/s12906-021-03221-5 (PMC7977270; doi:10.1186/s12906-021-03221-5)

**Fig. 1**

**a) WB**

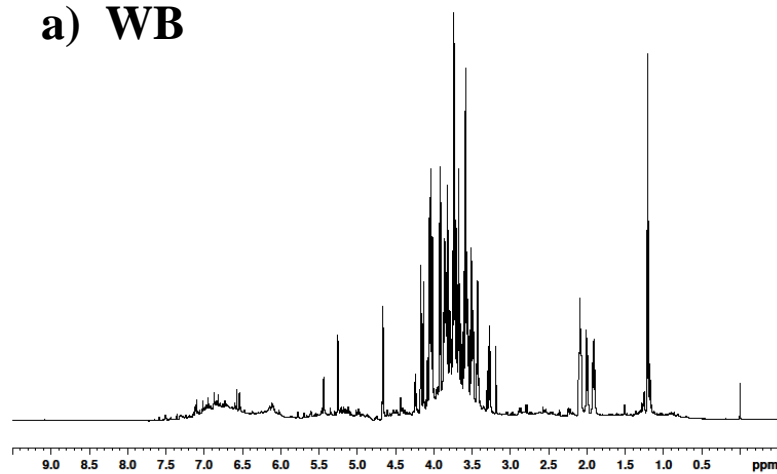

**b) WC**

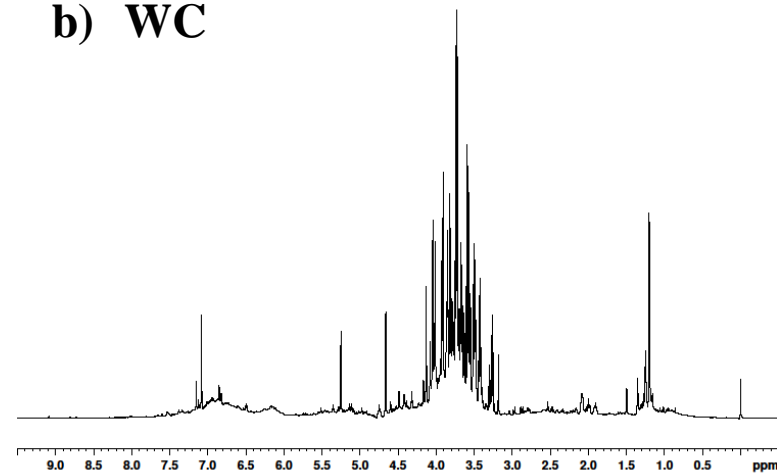

**c) WD**

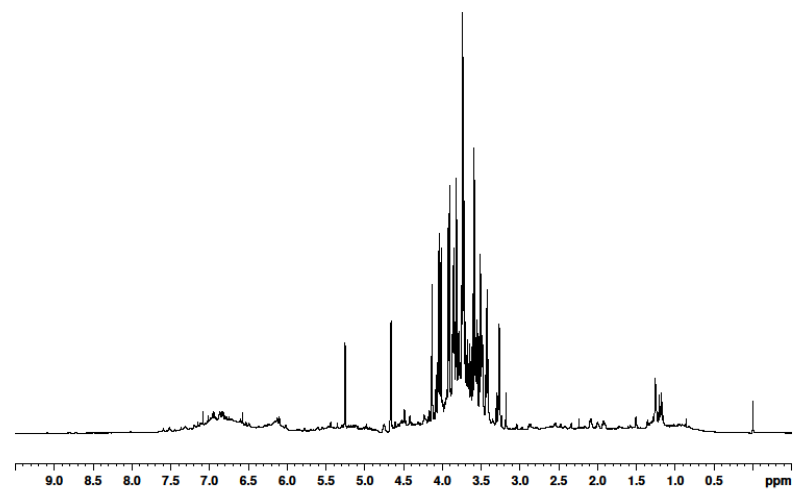

Supplement: Supplementary file 1 — Additional file 1: Fig. S1. Representative 1H NMR plots for seasonal differentiation. Individual plots are representative 1H NMR spectrum of the guava hydroalcoholic extract prepared from leaves collected from Shirwal (W region) in. a) season B (May 2013); b) season C (October 2013); c) season D (March 2014). [file 12906_2021_3221_MOESM1_ESM.pdf]

**Fig. 2**

**a) WD**

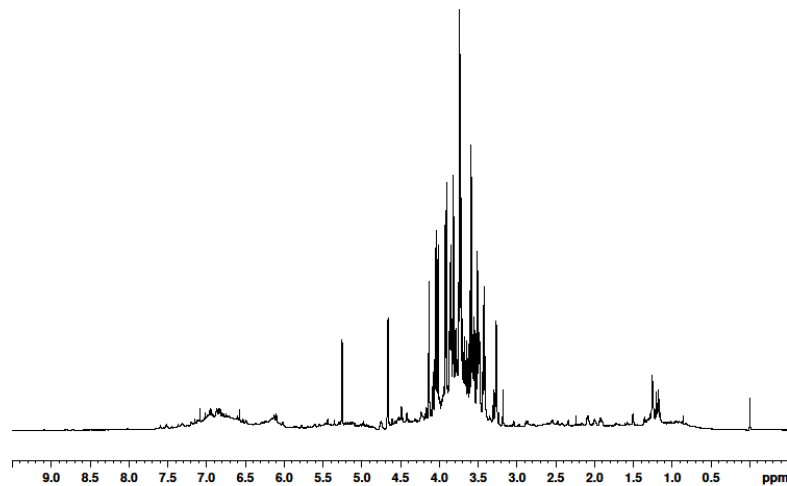

**b) RD**

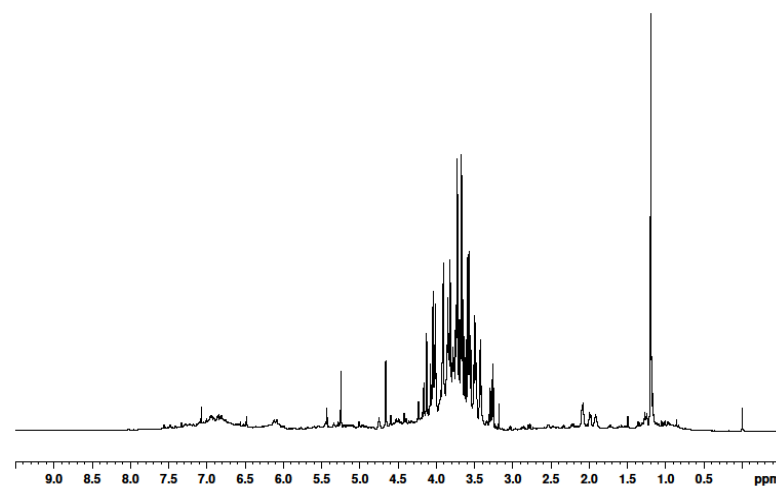

**c) DaD**

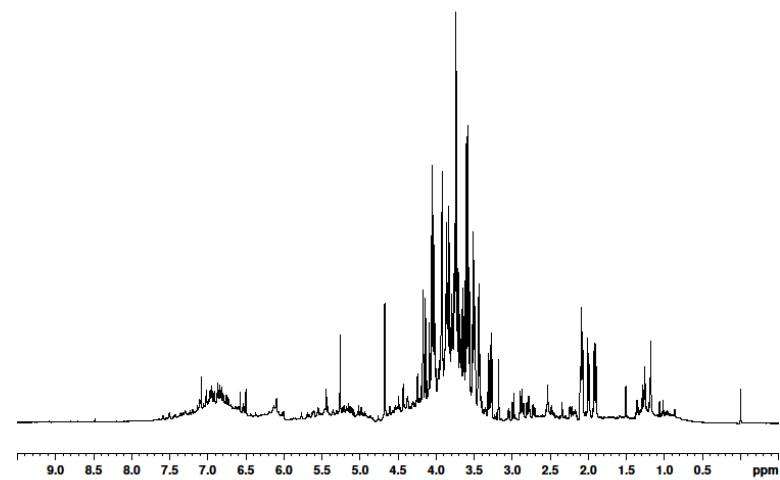

Supplement: Supplementary file 2 — Additional file 2: Fig. S2. Representative 1H NMR plots for regional differentiation. Individual plots are representative 1H NMR spectrum of the guava hydroalcoholic extract prepared from leaves collected in season D (March 2014) from. a) Shirwal (W region); b) Rahata (R region); c) Dapoli (Da region). [file 12906_2021_3221_MOESM2_ESM.pdf]

Fig. 3

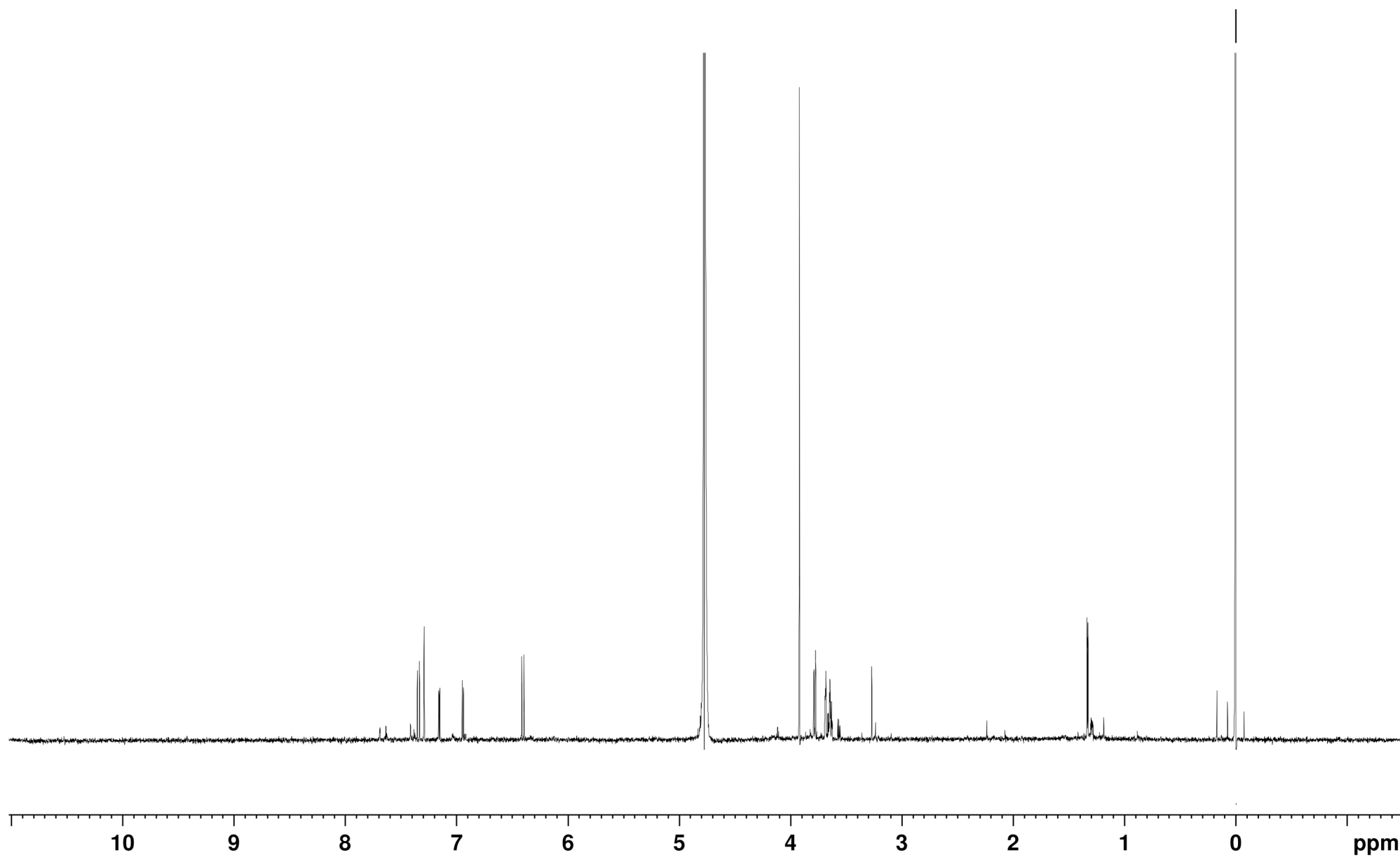

Supplement: Supplementary file 3 — Additional file 3: Fig. S3. 1H NMR plot acquired for quercetin. [file 12906_2021_3221_MOESM3_ESM.pdf]

Fig. 4

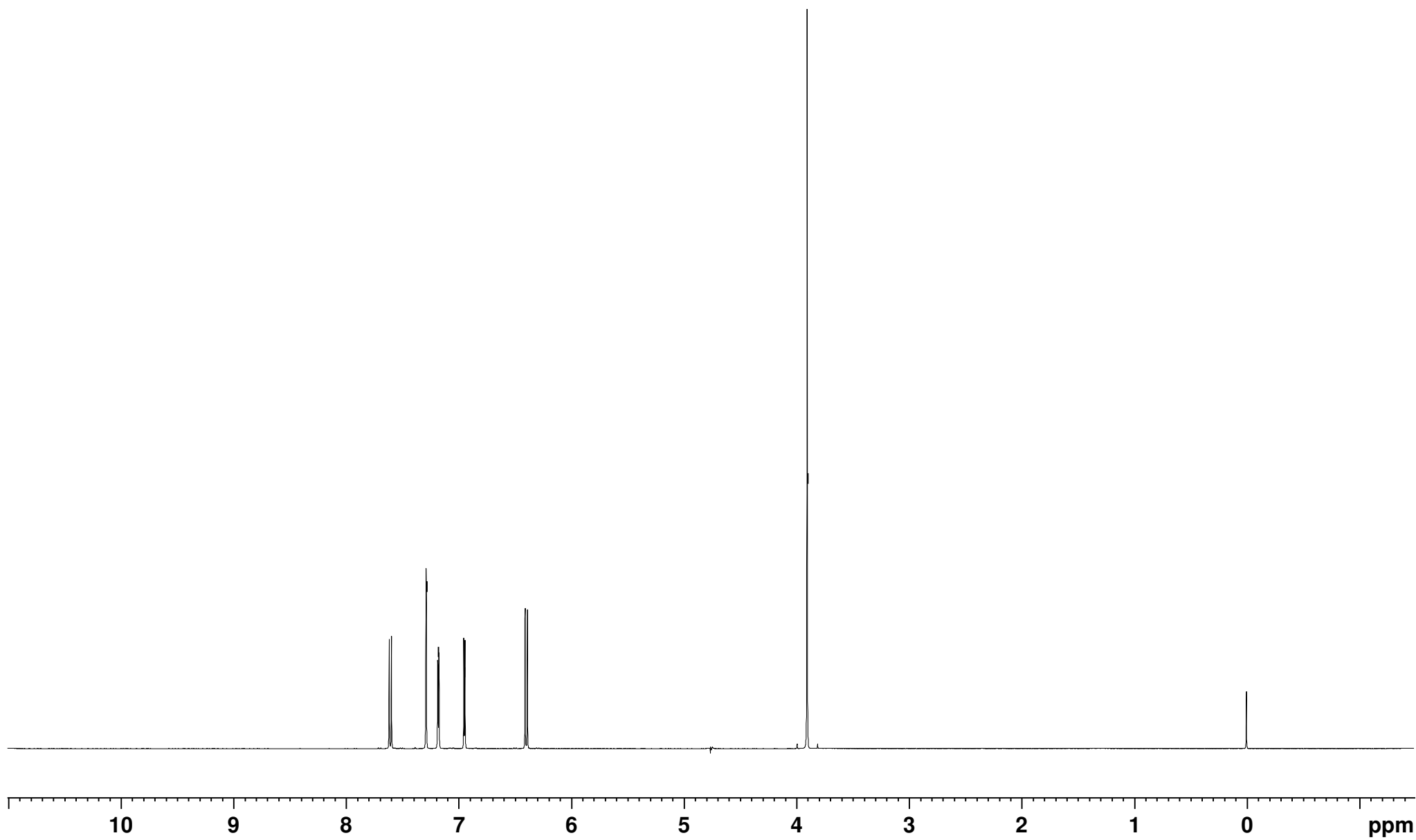

Supplement: Supplementary file 4 — Additional file 4: Fig. S4. 1H NMR plot acquired for ferulic acid. [file 12906_2021_3221_MOESM4_ESM.pdf]

Fig. 5

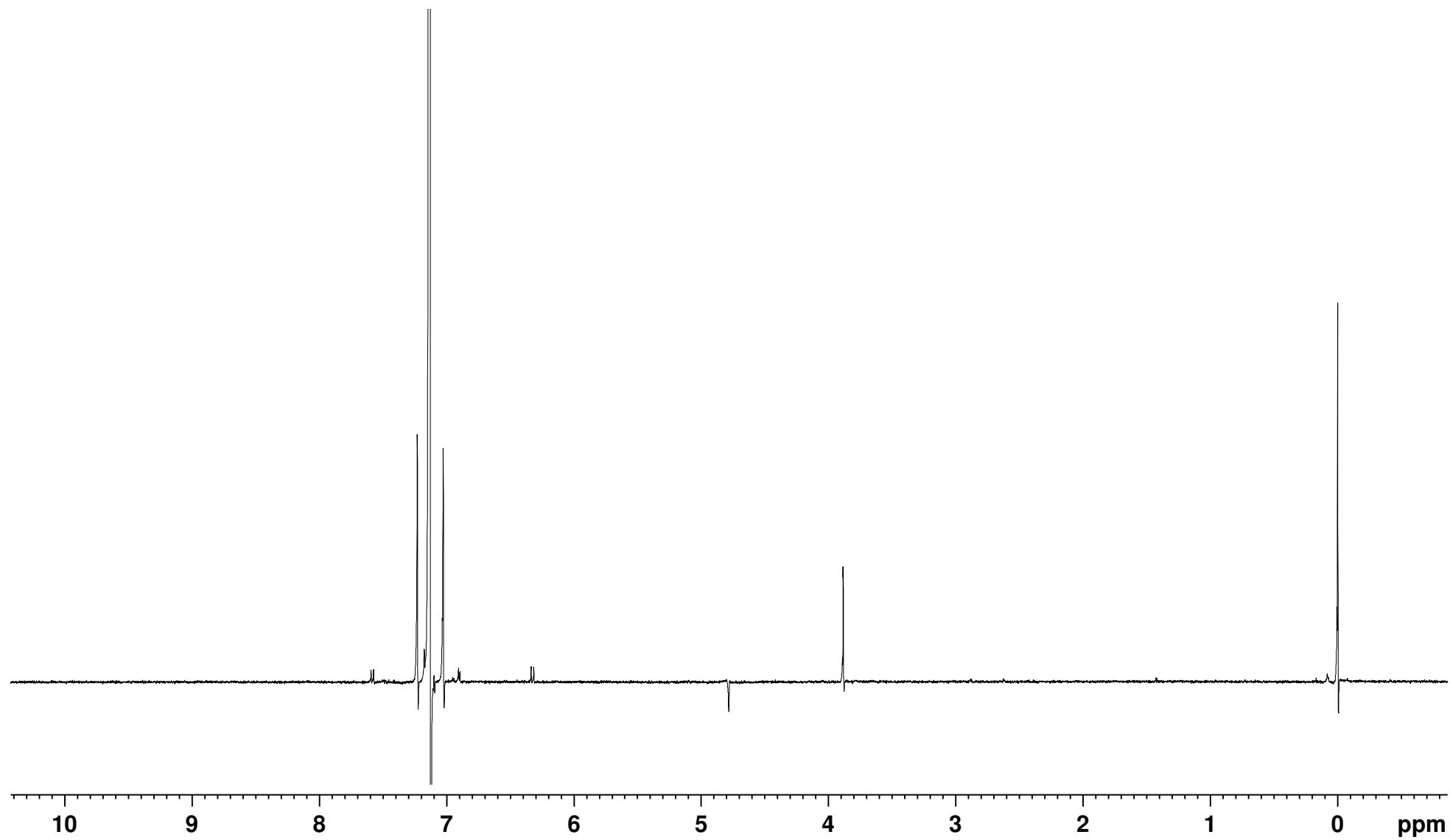

Supplement: Supplementary file 5 — Additional file 5: Fig. S5. 1H NMR plot acquired for gallic acid. [file 12906_2021_3221_MOESM5_ESM.pdf]
