# Supplementary material for: 1H nuclear magnetic resonance-based metabolite profiling of guava leaf extract: an attempt to develop a prototype for standardization of plant extracts
Source: BMC Complement Med Ther. 2021 Mar 18;21:95. doi: 10.1186/s12906-021-03221-5 (PMC7977270; doi:10.1186/s12906-021-03221-5)

Fig. 6

a) Fragmentation pattern for Quercetin

Event#: 10 Product Ion Scan(E+) Precursor: 303.20 CE:-25.0 Ret. Time : [14.183-

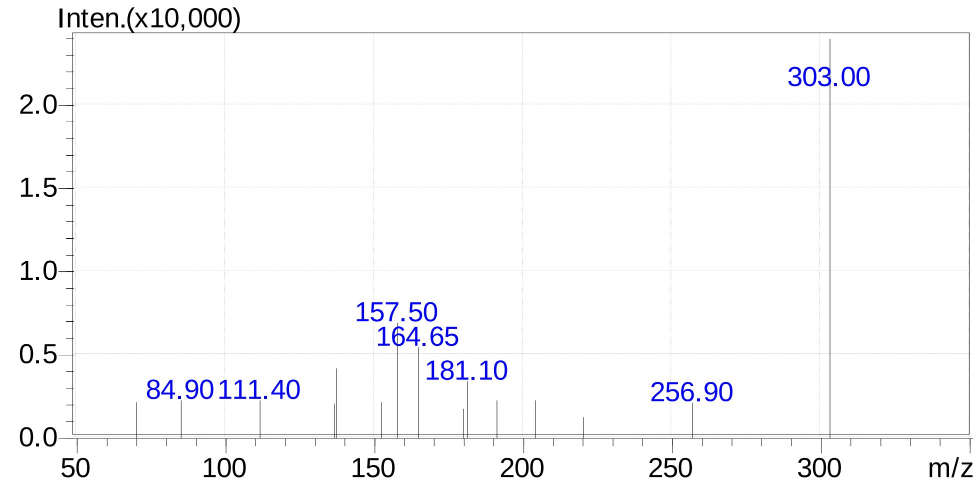

b) Fragmentation pattern for Gallic acid

Event#: 15 Product Ion Scan(E+) Precursor: 171.10 CE:-35.0 Ret. Time : [8.774-

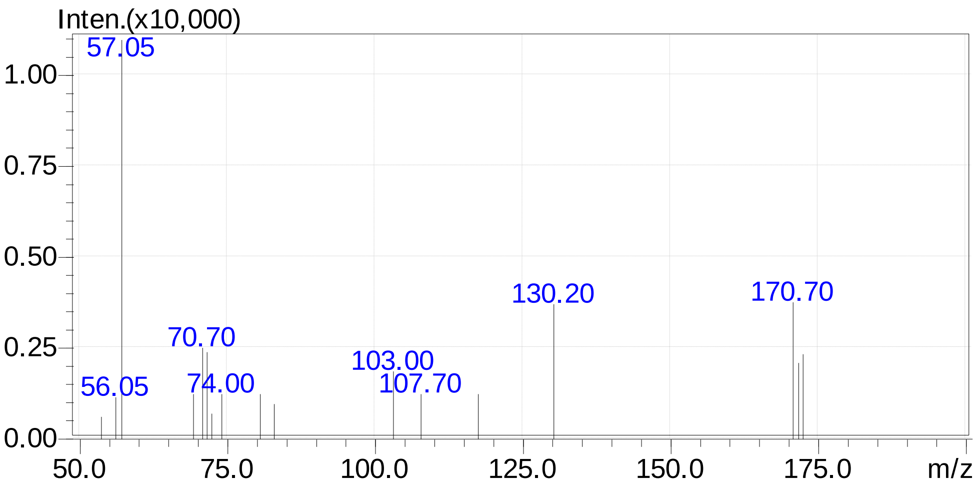

Supplement: Supplementary file 6 — Additional file 6: Fig. S6. Fragmentation patterns for compounds identified by LC-MS/MS in extract WB. W: Leaves collected from Shirwal region; B: May 2013 collection. [file 12906_2021_3221_MOESM6_ESM.pdf]
