# Supplementary material for: 1H nuclear magnetic resonance-based metabolite profiling of guava leaf extract: an attempt to develop a prototype for standardization of plant extracts
Source: BMC Complement Med Ther. 2021 Mar 18;21:95. doi: 10.1186/s12906-021-03221-5 (PMC7977270; doi:10.1186/s12906-021-03221-5)

Fig. 7

a) Fragmentation pattern for Quercetin

Event#: 9 Product Ion Scan(E+) Precursor: 303.20 CE:-35.0 Ret. Time : [13.919-

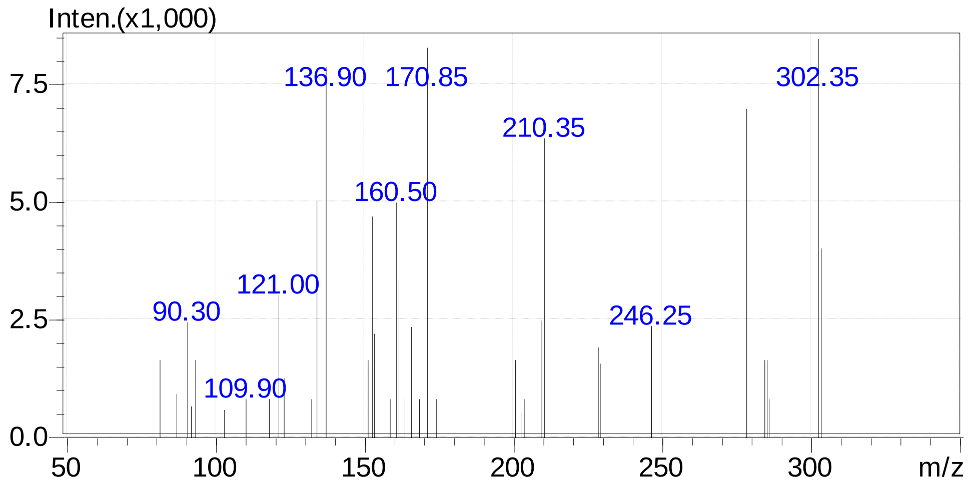

b) Fragmentation pattern for Ferulic acid

Event#: 6 Product Ion Scan(E+) Precursor: 195.05 CE:-35.0 Ret. Time : [4.672->6.213]-

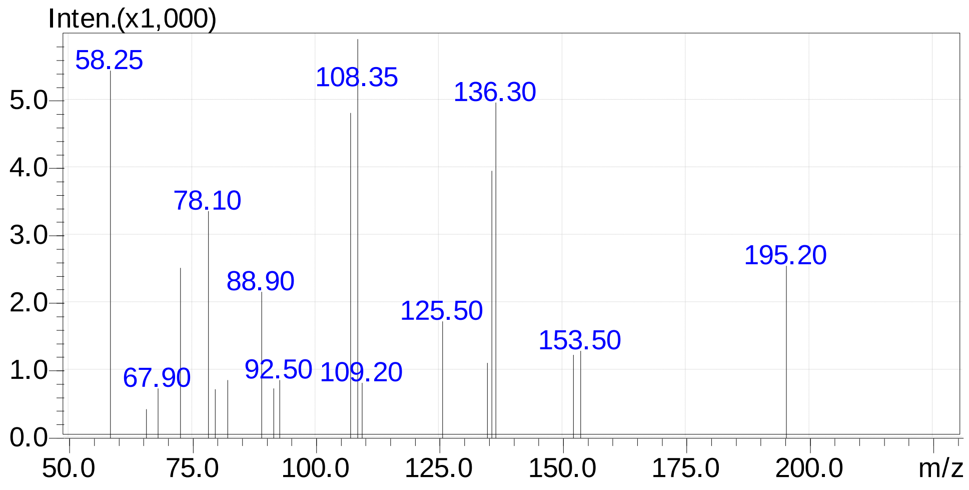

c) **Fragmentation pattern for Gallic acid**

Event#: 17 Product Ion Scan(E+) Precursor: 171.10 CE:-15.0 Ret. Time : [8.737-

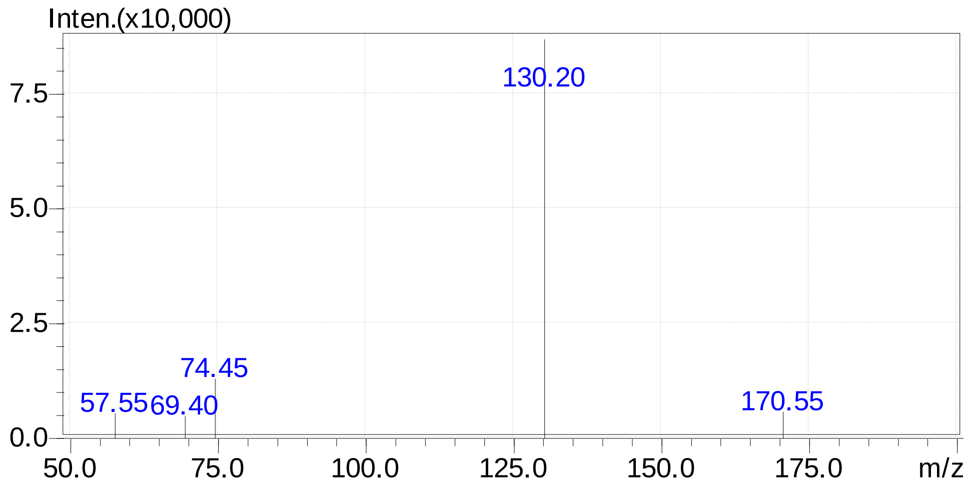

Supplement: Supplementary file 7 — Additional file 7: Fig. S7. Fragmentation patterns for compounds identified by LC-MS/MS in extract WC. W: Leaves collected from Shirwal region; C: October 2013 collection. [file 12906_2021_3221_MOESM7_ESM.pdf]
