# Supplementary material for: 1H nuclear magnetic resonance-based metabolite profiling of guava leaf extract: an attempt to develop a prototype for standardization of plant extracts
Source: BMC Complement Med Ther. 2021 Mar 18;21:95. doi: 10.1186/s12906-021-03221-5 (PMC7977270; doi:10.1186/s12906-021-03221-5)

Fig. 8

a) Fragmentation pattern for Quercetin

Event#: 9 Product Ion Scan(E+) Precursor: 303.20 CE:-35.0 Ret. Time : [13.731-

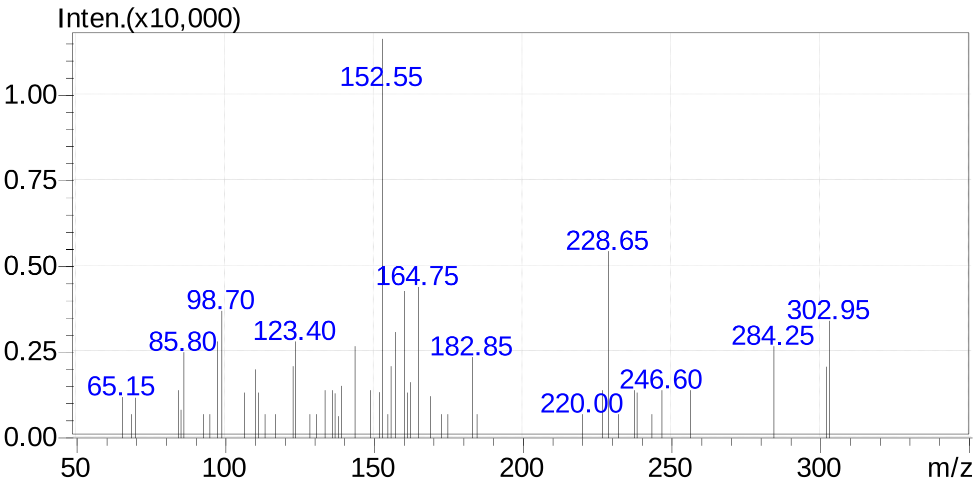

b) Fragmentation pattern for Ferulic acid

Event#: 8 Product Ion Scan(E+) Precursor: 195.05 CE:-15.0 Ret. Time : [4.398->6.468]-

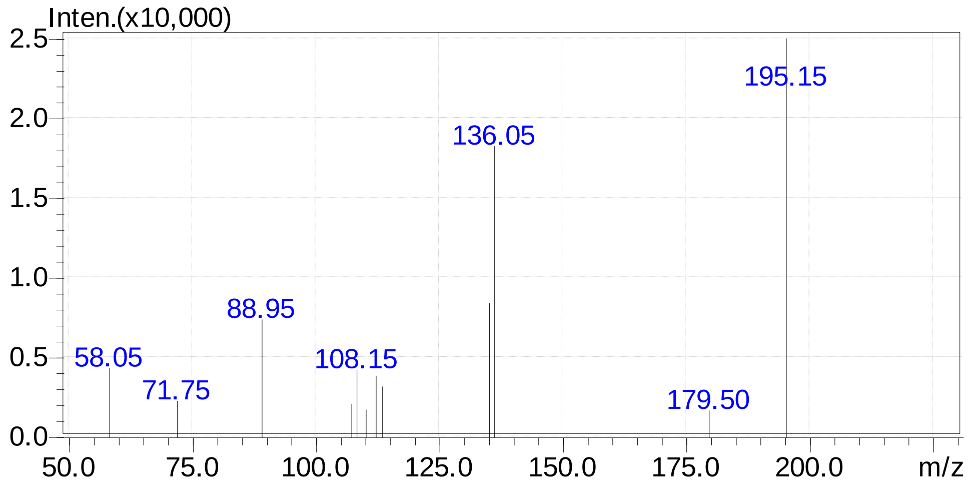

Supplement: Supplementary file 8 — Additional file 8: Fig. S8. Fragmentation patterns for compounds identified by LC-MS/MS in extract WD W: Leaves collected from Shirwal region; D: March 2014 collection. [file 12906_2021_3221_MOESM8_ESM.pdf]
