# Supplementary material for: 1H nuclear magnetic resonance-based metabolite profiling of guava leaf extract: an attempt to develop a prototype for standardization of plant extracts
Source: BMC Complement Med Ther. 2021 Mar 18;21:95. doi: 10.1186/s12906-021-03221-5 (PMC7977270; doi:10.1186/s12906-021-03221-5)

Fig. 9

a) Fragmentation pattern for Quercetin

Event#: 1 Product Ion Scan(E+)   Precursor: 303.00 CE:-35.0   Ret. Time : [14.153-

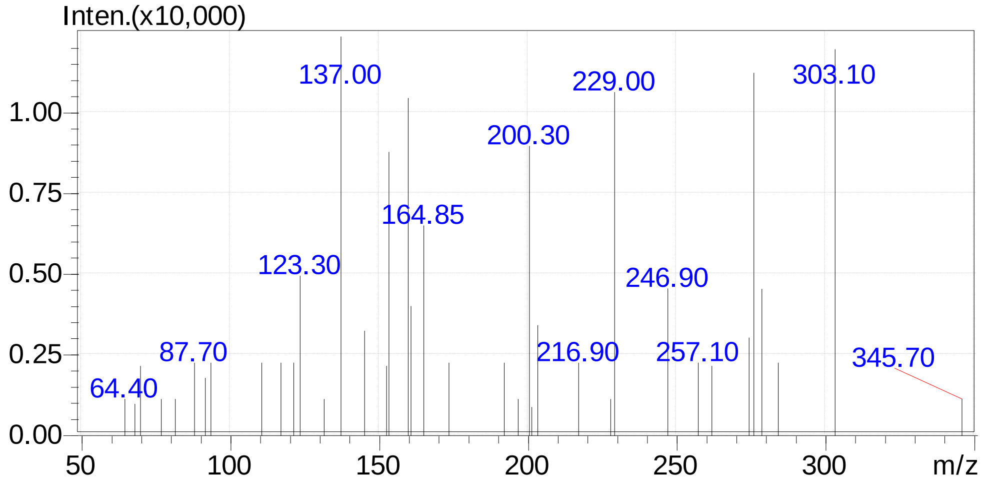

b) Fragmentation pattern for Ferulic acid

Event#: 7 Product Ion Scan(E+)   Precursor: 195.05 CE:-25.0   Ret. Time : [4.748->6.928]

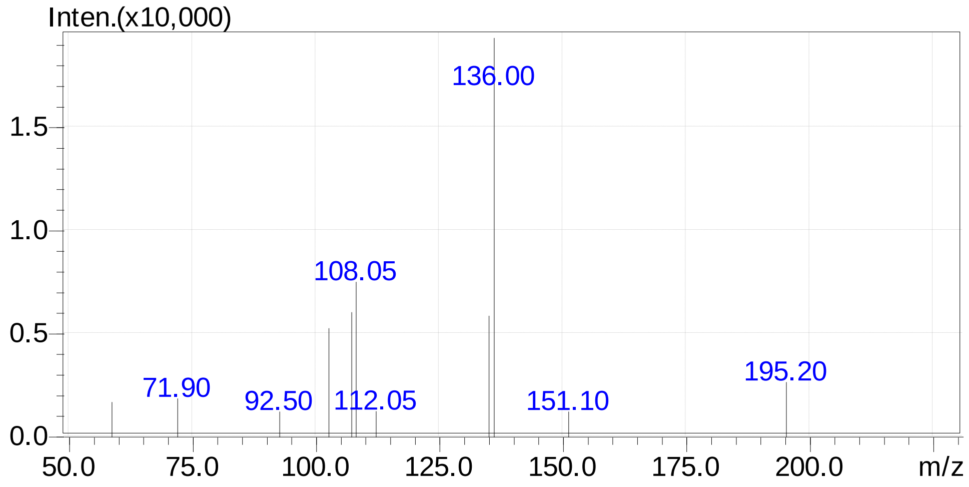

c) Fragmentation pattern for Gallic acid

Event#: 17 Product Ion Scan(E+) Precursor: 171.10 CE:-15.0 Ret. Time : [8.286-

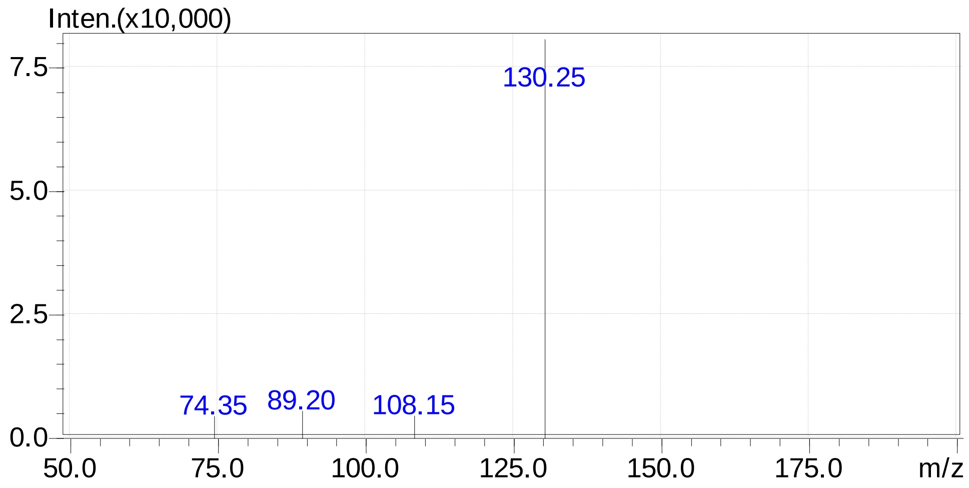

Supplement: Supplementary file 9 — Additional file 9: Fig. S9. Fragmentation patterns for compounds identified by LC-MS/MS in extract RD. R: Leaves collected from Rahata region; D: March 2014 collection. [file 12906_2021_3221_MOESM9_ESM.pdf]
